# Lean Wrought Magnesium Alloys

**DOI:** 10.3390/ma14154282

**Published:** 2021-07-31

**Authors:** Nikolaus P. Papenberg, Stefan Gneiger, Peter J. Uggowitzer, Stefan Pogatscher

**Affiliations:** 1 LKR Light Metals Technologies Ranshofen, Austrian Institute of Technology, A-5280 Ranshofen, Austria; stefan.gneiger@ait.ac.at; 2 Chair of Nonferrous Metallurgy, Montanuniversität Leoben, A-8700 Leoben, Austria; peter.uggowitzer@mat.ethz.ch (P.J.U.); stefan.pogatscher@unileoben.ac.at (S.P.)

**Keywords:** magnesium alloys, dilute alloys, lean alloys, literature review, overview

## Abstract

Lean magnesium alloys are considered attractive candidates for easy and economical hot forming. Such wrought alloys, defined here as materials with a maximum alloying content of one atomic or two weight percent, are known to achieve attractive mechanical properties despite their low alloy content. The good mechanical properties and the considerable hardening potential, combined with the ease of processing, make them attractive for manufacturers and users alike. This results in potential uses in a wide range of applications, from rolled or extruded components to temporary biomedical implants. The characteristic behavior of these alloys and the optimal use of suitable alloying elements are discussed and illustrated exemplarily.

## 1. Introduction

Magnesium is widely acknowledged as the lightest structural metal and is therefore known as being a good candidate for applications where product weight plays an important role [1]. Many such potential applications are structural parts, which are usually produced by forming processes, and are characterized by improved homogeneity, reduced defect size and enhanced mechanical properties, compared to castings.

Nevertheless, more than 90% of Mg parts in use are produced by casting [2], showing that the use of Mg in structural applications and the utilization of wrought products in general is still lacking.

While it has been demonstrated repeatedly that Mg wrought alloys can show a wide spectrum of mechanical, chemical and physical properties [3,4,5], multiple reasons for their limited use can be found. Particularly noteworthy is the small number of industrially available alloying compositions, lack of stock material, the use of high-priced alloying elements, and last but not least, a lack of practical knowledge regarding material processing and utilization [6,7,8]. In particular, the experience deficit highlights the mismatch of cast and wrought products. This difference is deeply rooted and amplified by the challenging forming behavior of Mg alloys, discouraging manufacturers and preventing widespread application.

Numerous attempts have been made to improve the forming behavior and performance of Mg wrought alloys by the adaptation of processing and alloying, producing mixed results. Nevertheless, steady progress has been made; nowadays, Mg products can be produced at high speeds [9] and can exhibit remarkable mechanical properties [10,11]. In recent years, alloying concepts using only low amounts of alloying elements have shown impressive results in a broad range of topics [4,12,13]. This design approach attempts to make full use of the alloying elements and tries to combine the processing and material response in the best possible way. Since this principle can be applied to various systems within the Mg alloy range, the possible applications are diverse and the topics of investigations are correspondingly wide ranged.

In the case of materials for structural applications at industrial scale, e.g., extruded profiles, the main drivers are low material and production costs. This can be realized with cheap alloying elements but, more importantly, with high production speeds [14]. The deformation behavior in multiple subsequent forming operations is also a crucial issue, i.e., in sheet forming. In other areas, such as bio-medical applications, corrosion behavior is the focus of interest.

This review aims to give a short overview of the topic ’lean wrought magnesium alloys’ by summarizing and discussing multiple relevant scientific publications in this field. For this study, the authors limited the selection of investigated works to publications using ternary and more complex Mg wrought alloys, with a combined alloying content lower than ~1 atomic percent (at.%) and ~2 weight percent (wt.%). As the focus of this review lies on the conventional processing of these alloys, works concerning production by severe plastic deformation (SPD) or powder metallurgy are not covered.

For ease of understanding, we first consider often used terms and definitions in Section 2 and basic aspects of alloy developments in Section 3. Various alloys and their applications are discussed in Section 4, and a brief conclusion is given in Section 5. For further clarification, a summary of the discussed alloys is provided in the Appendix B and Appendix C, listing the chemical compositions and the mechanical properties, including the corresponding processing parameters.

## 2. Terms and Definitions

When searching for scientific literature on the topic of ’low alloyed Mg alloys’ it becomes apparent that this is a diffuse subject. There are no clear definitions of the alloy specifications, and the terms describing them are constantly changing. For a better understanding, we briefly discuss the commonly used terms in the following paragraphs.

When adding small amounts of alloying elements to already existing materials, the term **micro-alloying** is often used. Typically, the added elements are kept below 1 wt.% to be called micro alloying [15]. This practice is well known and used for an abundance of materials to improve specific material properties and the processing behavior. Typical applications for Mg alloys are reductions of the texture by the addition of rare earth elements (RE/REE) [16], the use of Ca and Y to enhance the oxidation stability [17,18] and Mn additions to improve the corrosion behavior [3]. As a micro alloyed material is not necessarily an alloy with low alloying content, this review will only deal with micro-alloyed materials that meet our definition of lean alloys.

The abbreviation **HSLA**, meaning ’High Strength Low Alloyed’ is used in ref. [19]. The described Mg alloys replicate the approach of increased material strength by grain refinement, known from the steel industry. This is achieved by the use of a small amount of alloying elements influencing the recrystallization and grain growth behavior during hot forming processes.

The terms **low alloyed**, **dilute** and **lean** all aim to describe Mg alloys, using only a small overall amount of added elements. The maximum amount of used alloying elements can, nevertheless, vary considerably, ranging from 0.16 wt.% [20] up to 3.82 wt.% [21]. The reason for this is the difficulty to define a strict range of the total amount of used additions for the term low alloyed. The term can be subjectively interpreted, as most wrought alloys are low alloyed when compared to casting alloys.

Despite these challenges, an attempt to define the term ’lean alloy’ was made in refs. [22,23], where the authors restricted themselves to alloys, using alloying elements up to a sum of 1 at.%. As these works concentrate on alloys using mainly light elements, e.g., Al and Ca, this seems reasonable. On the other hand, this definition proves insufficient when applied to alloys with heavy elements, e.g., REE.

Other possible weighting or limiting definitions only seem to increase the complexity of this problem and cannot, therefore, be the preferred approach. While at.% is often used in studies on alloy development, phase descriptions and element solubility, we use wt.% as a reference system, as it is the most common way to describe Mg alloys and improves the ease of comparability significantly. Nevertheless, tables containing the chemical compositions of the discussed alloys in wt.% and at.% can be found in Appendix B.

In this review, we limit ourselves to conventionally processed wrought alloys with a maximum alloying content of ~1 at.% or ~2 wt.%. Additionally, the selected alloys are required to have at least two alloying elements. This is done to narrow the range of the discussed alloys down to a reasonable level, while still maintaining a broad range of possible alloys and processing schemes. In this way, a more focused approach on this interesting and diverse topic is made possible.

**Figure 1:**
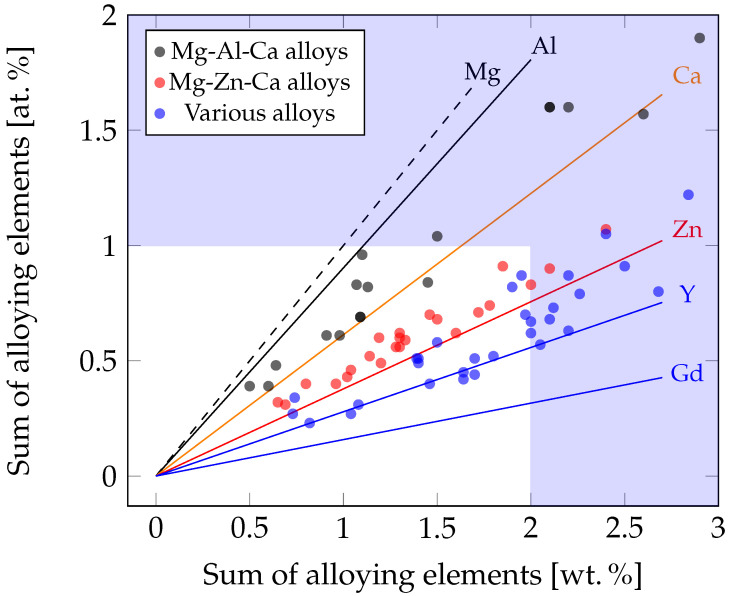
Effect of alloying elements and content on the alloys discussed in this work (limits of ~1 at.% and ~2 wt.%). Dots mark individual alloys, while lines show the trend of binary Mg alloys with the indicated element.

For better understanding, an overview of the amount of alloying elements in wt.% and at.% is shown in Figure 1. There, the alloys discussed in this work as well as binary alloys and the chosen limitations are depicted. Some alloys outside the limitations are included, as they are used for comparison within the investigated works.

As the discussed materials are within a narrow range, regarding their chemical composition, the nomenclature consists of the alloying elements and their corresponding amount in wt.%, e.g., Mg-1.0Zn-0.5Ca or Mg-0.3Al-0.2Ca-0.5Mn. While a little laborious to read, this description allows for an easy comparison of the alloys and directly shows the relevant chemical composition.

## 3. Basic Aspects of Alloy Development

The development and improvement of Mg alloys is a very complex subject that combines information from a wide range of multiple metal physic phenomena [24]. It is important to realize that the interaction of microstructural features is an intrinsic feature. Slight changes of alloying content can influence the solidus temperature, precipitation behavior, possible activation of slip systems or oxidation behavior. Therefore, only a fragmentary introduction, which is furthermore trimmed to the use of lean alloys, can be given here. It is further essential to keep in mind that the microstructural properties and effects during the production process (i.e., raised temperatures) and behavior in use can differ greatly.

**Figure 2:**
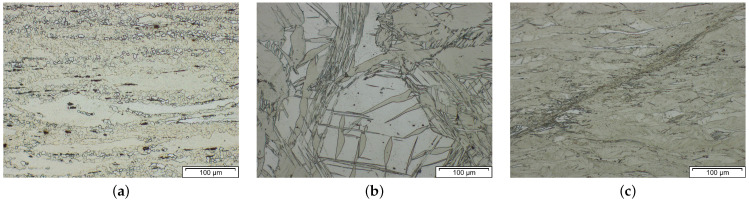
Examples of microstructural features common in deformed Mg alloys. Pictures show a Mg–Al–Ca lean alloy produced at LKR Light Metals Technologies Ranshofen in an as-forged state. (**a**) Necklace structure; (**b**) twins; (**c**) shear.

As the main aim of Mg wrought alloys development is their production by forming operations, such as extrusion or rolling, it is imperative to be aware of their challenging deformation behavior. Mg alloys, including lean variants, can show a broad variety of microstructural features during processing, dependent on alloy and forming parameters, as shown exemplarily in Figure 2. The original microstructure resulting from the casting process is deformed and the grains may exhibit twinning and/or areas of dislocation slip. When the temperature is sufficiently high, recrystallization becomes a common feature. It usually starts during the forming process and can occur dynamically (during forming), meta-dynamically (during cooling processes) and statically (during heat treatments). The nucleation points for new grains are manifold, e.g., twins, grain boundaries and particles. Dynamic recrystallization behavior during forming often results in a broad grain size distribution. The combination of newly formed small grains and original deformed microstructure is typically called a necklace structure [25,26].

The material’s forming ability is controlled by the hexagonal crystal structure and the ensuing large differences in the critical resolved shear stress (CRSS) of the available slip and twinning systems. Deformation is mostly accommodated by dislocation movement on the basal plane or by twinning, but neither mechanism provides the necessary amount of slip systems for arbitrary deformation; therefore, deformation at room temperature (RT) is impeded considerably. Consequently, most forming operations in which the material is forced to accommodate large strains take place at elevated temperatures. Here, the activation of the prismatic and pyramidal slip systems is advantageous, which is made possible by the converging CRSS values.

The differences in CRSS between the deformation mechanisms [4] also amplifies the texture development during directional forming processes (and subsequent heat treatments), leading to pronounced basal textures in various investigated Mg sheets and profiles [13,27]. Such a strong structural anisotropy is reflected in the mechanical properties, e.g., the ratio of tensile yield strength (YS) and compressive yield strength (YSc) [28], as well as in the forming behavior of these products.

As the deformation behavior and the texture development are intrinsically linked, it is not surprising that extensive studies are being conducted regarding this aspect. Next to process adaptations [13], e.g., cross rolling, the alloy customization by (micro-) alloying with, for example, REE or Ca, is an accepted practice for texture reduction and enhanced forming behavior [16,29,30,31,32]. In particular, the combination of Zn and Ca is known to improve the forming behavior of various Mg alloys. While effects on texture weakening and increased non-basal slip by these elements are well known, Basu et al. [33] recently investigated this effect in detail, using micro-pillar indentation at RT. It was found that even dilute additions of Zn and Ca lower the stacking fault energy drastically, thereby promoting pyramidal <c+a> dislocations and reducing the mobility of basal slip, enabling a more homogeneous material deformation. please confirm if here is right. NP: This is correct.

In the case of lean alloys, further improvement in the deformation behavior is achieved by a reduction in solute drag, as these materials evidently feature a reduced amount of soluble alloying elements. However, as this effect is dependent on the solubility potential of the alloying elements used, no general description can be given here [34,35].

As material strength is the feature dominating the potential fields of application for structural materials, the possible means and design principles of increasing mechanical strength are briefly discussed below. In the case of dilute alloys, only grain refinement and precipitation hardening are relevant, as solid solution strengthening is not a viable option.

**Figure 3:**
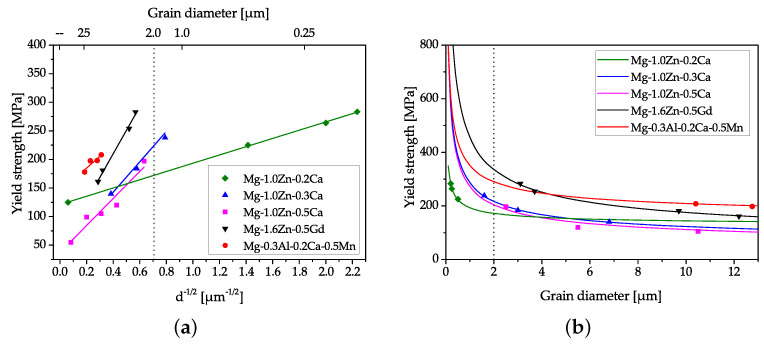
Hall–Petch slopes of multiple Mg lean alloys (**a**), as well as the calculated rise in YS values with decreasing grain size in (**b**). Experimental data were taken from refs. [28,40,41,42,43], and further information is given in Table A3.

**Grain refinement** is well known for its positive influence on the strength and deformation behavior of practically all metallic materials, particularly for Mg. This property is important for an improved performance during forming operations [36] and is often regarded as the main strengthening mechanism in dilute Mg alloys [4]. The effect of grain refinement is typically described via the Hall–Petch relationship, which predicts a significant rise in YS for fine-grained structures. This formulation is based on the assumption of an isotropic poly-crystalline material; therefore, the application on wrought Mg alloys with their tendency for texture formation might give diffuse values, as the Hall–Petch relationship shows a dependence on texture and strain [37,38,39,40]. Additionally, a decline in the ascent of the Hall–Petch slope (k${}_{y}$) is reported for Mg alloys with a grain diameter (*d*) smaller than the range of 2 to 3 μm [39]. As many Mg parts are investigated in F-temper, further uncertainty is added for the evaluation of the Hall–Petch relationship by the interaction of deformed and recrystallized microstructures. For comparison, an extract of the Hall–Petch behavior for some Mg alloys is given in Figure 3 and Table A3.

The effect of strengthening by grain refinement is only pronounced at small grain sizes, easily visible in Figure 3b. As such small grains cannot be produced by conventional casting, forming processes with a high degree of deformation are necessary. The most common industrial processes to achieve grain refinement are extrusion or rolling. In cases where even finer grain sizes are needed, processes of severe plastic deformation, e.g., equal channel angular pressing (ECAP), high pressure torsion (HPT) or multi axial forging (MAF), might be used [44].

A downside of fine-grained materials is the strong grain growth tendency, which poses a major challenge to subsequent processing or use at raised temperatures. To mitigate this effect, the Zener drag provided by precipitating phases (i.e., dispersoids and precipitates) has been used with good success [28].

**Precipitation hardening** is well known from the use in Al alloys and is the main reason for the success of the 2xxx, 6xxx and 7xxx series [45]. While known in Mg alloys as well, the use of precipitation hardening is not that far spread [46], and process chains like those common in Al wrought alloys are few.

As shown by Nie [47], precipitation hardening is possible for several Mg alloying systems, but it is important to consider the positioning of the precipitates. The best hardening effects are shown by particles that impede basal slip, as it is the most easily activated slip system of the hexagonal Mg crystal, i.e., the most important for the deformation process at RT. As an example, Al–Ca-rich Guinier–Preston (G.P.) zones in lean Mg–Al–Ca alloys may be mentioned here, which enable a considerable increase in strength [23]. The precipitation of hardening phases can either happen dynamically during processing, where the forming phases are known to delay the dynamic recrystallization [26] and stabilize grain boundaries [48], or can be facilitated with adequate heat treatments, where precipitation can be controlled more easily. Due to the temperatures used, especially during solution heat treatment, an increase in the grain size is often unavoidable, but can be reduced by already existing phases, e.g., dispersoids. Additionally, intermetallic phases (IMP) can play a major role in the corrosion behavior, which is an important aspect in biomedical applications, and is further described in Section 4.2.

**Figure 4:**
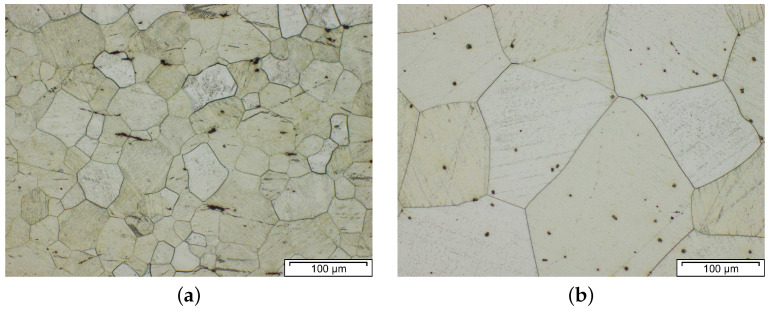
Comparison of the grain size within a forged and solution heat-treated low-alloyed Mg alloy containing REE. Showing the microstructure (**a**) with Zr additions (~0.4 wt.%) and (**b**) without Zr. Experiments performed at LKR Light Metals Technologies Ranshofen.

While **dispersoids** are neglectable with respect to particle hardening, they can play an important role in the processing behavior. These phases, with varying chemical compositions, can either be formed during casting or preferably during subsequent heat treatment/processing steps, where a regulation of particle size and distribution can be achieved by appropriate heat treatment parameters. The dispersoids counteract the grain growth, due to their Zener pinning pressure [49], which increases with the reduced particle size and increasing volume fraction. An example of the pinning effect is given in Figure 4, where two alloys with and without Zr dispersoids are compared. In Al free Mg alloys, Zr is often used as the dispersoid forming element, while in Mg–Al alloys, the use of Al–Mn intermetallics is appropriate [23]. The Zr additions are also effective as grain refiners during casting, enabling the production of fine-grained stock materials [50].

## 4. Mg Alloys with a Low Alloying Content

This section is divided into three parts, analyzing the most-used alloying systems, namely Mg–Al–Ca (Section 4.1) and Mg–Zn–Ca (Section 4.2) as well as descriptions of various alloy compositions in Section 4.3. An overview of the discussed alloys is compiled in the appendices, where the chemical compositions (Appendix B) and mechanical properties (Appendix C) are listed.

**Figure 5:**
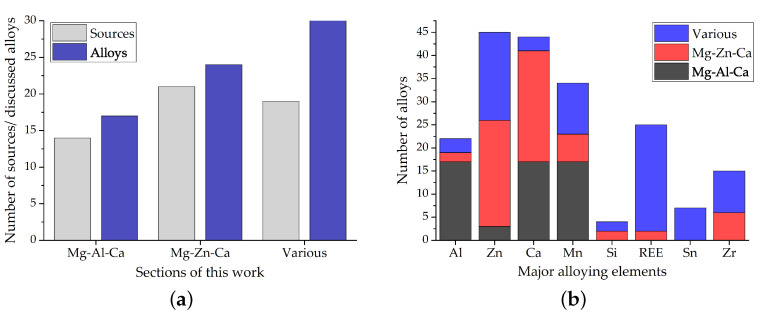
Overview of (**a**) the number of publications and alloys discussed in this work and (**b**) the number of alloys separated by major alloying elements found in this review.

While there are almost unlimited possibilities for variations in the chemical composition of lean alloys, it becomes apparent from examining the published literature that there is a trend toward the use of Mg–Al and Mg–Zn alloys; see Figure 5. This is not surprising, as these systems are the primarily produced and investigated alloying systems for Mg wrought alloys. While (micro-) alloying with REE is a common practice for Mg alloys, lean alloys using RE additions are not as common as expected. This might be partly because of the chosen limiting values of ~2 wt.% of alloying elements in the investigated materials, which restricts the use of heavy elements accordingly.

Rather surprising is the high number of Ca containing alloys, which can nevertheless be well explained. Ca is increasingly used as a secondary element in both main alloying systems, Mg–Al and Mg–Zn, as it can facilitate various interesting properties, such as grain refinement during casting [51], increased oxidation stability [52,53], potential of IMP phase formation and improved creep resistance [54,55,56]. The most important feature for wrought alloys might be the promotion of pyramidal slip during deformation [57] and the associated reduction in texture intensity [58,59,60].

The number of sources and the number of discussed alloys, divided into the sections used in this work, are shown in Figure 5a. While the number of alloys in Section 4.1 and Section 4.2 are restricted by using the combination of two major alloying elements, Al–Ca and Zn–Ca, respectively, the number of references and alloys is comparable to Section 4.3, ’Alloys containing various Elements’. This highlights the focus that is given to these alloying systems throughout the scientific literature. Additionally, it can be noted that the cited references in Section 4.1 and Section 4.2 are more focused on in in-depth investigations of potential alloys, when compared to Section 4.3, where a general discussion of multiple alloys per publication is more common. This is evident from the smaller number of sources, compared to the number of discussed alloys.

Using the overview of the investigated alloys given in Figure 5b, some interesting facts about the composition of Mg alloys in general can be noted. The combination of Mg–Al and REE [61] as well as Zr [62] is avoided in wrought alloys, as Al is known to form high melting IMPs with these elements, which cannot be dissolved in subsequent processing steps. The combination of Mg–Zn and REE and/or Zr, on the other hand, is a well-established practice and used to increase the overall performance of these alloys [61,62]. The fact that Ca can be considered an alternative for REE additions [63,64] is visible as well, as the combination of Ca and REE in the same section is minimal.

### 4.1. Alloys Containing Aluminum and Calcium

Wrought alloys of the Mg–Al–Ca (AX) system have been intensively investigated in recent years. These alloys make optimal use of low-cost alloying elements and have shown their ability to achieve strengths [10,11] or show favorable forming behavior [9,24]. The optimal combination of these two properties has not yet been found, but alloy developments are steadily advancing toward this goal. Extruded material has been especially well investigated. The processing of such alloys by extrusion is attractive because of the high degree of deformation possible and the almost isotherm forming temperatures at moderate extrusion rates. Nevertheless, the complexity of this process increases markedly at high forming rates and with the production of hollow profiles and/or complex cross sections. Therefore, round bars are used as test geometry in most publications investigating extrusion processes [27].

**Figure 6:**
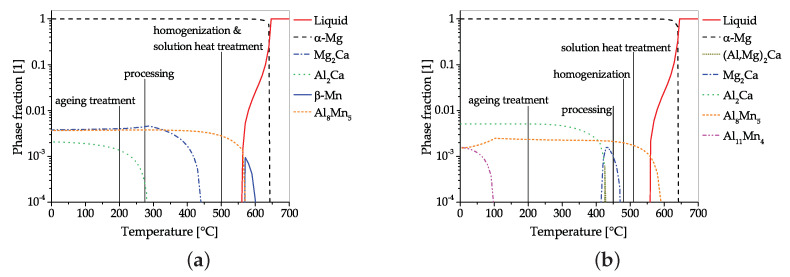
Comparison of CALPHAD calculations of Mg–Al–Ca alloys, including the used processing temperatures. Calculations were made using the alloy compositions given in refs. [23,71]; for further information, see Appendix A.2. (**a**) Mg-0.3Al-0.3Ca-0.4Mn; (**b**) Mg-0.6Al-0.3Ca-0.3Mn.

While high strength, up to 450 MPa UTS, is an attractive feature for extruded Mg–Al–Ca–Mn products [11,65], the achievable RAM speed of these alloys was considered insufficient for widespread industrial application. The aim to increase the extrusion speed was the first step toward the development of low alloyed Mg–Al–Ca alloys, using complex processing chains. Based on works reporting an increase in extrusion speed with reduced alloying elements [66,67,68,69], Nakata et al. [9,40] presented a heat treatable (T5) Mg-0.3Al-0.2Ca-0.5Mn alloy, which can be extruded with die-exit speeds of up to 60 m min${}^{-1}$, using extrusion temperatures in the range of 350 to 500 ${}^{\circ }$C. In a subsequent work [70], the effect of the Mn content (range 0 to 0.84 wt.%) on the processing behavior, heat treatment and mechanical properties was investigated. Of the tested compositions, the Mg-0.3Al-0.3Ca-0.4Mn alloy showed the highest tensile properties, reaching 264 MPaUTS and 220 MPaYS in a peak aged state (T6). Further development was aimed at an enhancement of the mechanical strength, which was achieved by slightly increasing the alloying content [71]. As these investigated alloys showed inconsistent age-hardening behavior, this strategy was adapted by thermodynamic calculations to achieve maximum age hardening in [22,23,72]. This culminated in the development of a Mg-0.6Al-0.3Ca-0.3Mn alloy, reaching a YS of 253 MPa and UTS of 277 MPa at peak hardness (T6), thereby showing a very high ratio of precipitation hardening effect onto the alloying content (YS/at.%) [23]. The adaptations made to the alloy composition and the processing are subsequently illustrated below with the help of CALPHAD calculations (Figure 6).

When comparing the processing and precipitation behavior of the alloys Mg-0.3Al-0.3Ca-0.4Mn [71] and Mg-0.6Al-0.3Ca-0.3Mn [23], using CALPHAD calculations, only slight overall differences in processing and alloying content are apparent. Both alloys aim to make use of the attractive age-hardening possibilities offered in the Mg–Al–Ca system, i.e., the precursor phases of Mg${}_{2}$Ca (C14) and Al${}_{2}$Ca (C15) [47]. To achieve peak hardness, both alloys are solutionized and age hardened (T6) after extrusion. As the high temperatures used for the solution heat treatment are known to facilitate unwanted grain growth, dispersoids are used as a retardant in both alloys. In this alloying system, the utilization of AlMn phases as grain boundary stabilization seems natural, as Mn is an often-used micro-alloying element, usually applied to increase the corrosion resistance by binding unwanted Fe. When looking at CALPHAD calculations (Figure 6) it can be seen that these Mn-containing phases already form during the casting process. As these calculations reflect the equilibrium state, the formation can be partially suppressed by appropriate casting processes and adequate cooling speeds. Accordingly, AlMn phases are either formed as coarse primary phases in the casting process itself or as precipitate during the subsequent homogenization heat treatment. Because of their high temperature stability, these AlMn dispersoids are present throughout the whole forming and heat treatment process, where their pinning effect retards dynamic recrystallization and hampers grain growth.

One obvious difference is the processing temperature, which takes place at much lower temperatures in ref. [71]. Nevertheless, the precipitation of C15 and C14 phases is possible in both alloys, which might also have a pinning effect on the microstructure, as reported by [26,48]. In both cases, the solution heat treatment (at ~500 ${}^{\circ }$C) is used to dissolve existing C14/C15 phases for the subsequent age hardening.

The aging treatment takes place at 200 ${}^{\circ }$C in both alloys, but the precipitating phases differ. In the Mg-0.3Al-0.3Ca-0.4Mn alloy, both C14 and C15 precursor phases are used for hardening, while the Mg-0.6Al-0.3Ca-0.3Mn uses only the Al${}_{2}$Ca precursor. While the amount of precipitating phases (∑ of C14 and C15) is comparable in both alloys (range 0.5 to 0.6% molar phase fraction), a distinctive difference in ΔYS is visible after age hardening. The YS of the Mg-0.3Al-0.3Ca-0.4Mn alloy increases by 46 MPa up to 156 MPa [71], while the Mg-0.6Al-0.3Ca-0.3Mn alloy reaches a YS of 253 MPa, which is an increase of 97 MPa [23]. This indicates the higher hardening potential of the Al${}_{2}$Ca precursor than that of Mg${}_{2}$Ca in the used T6 process.

Another in-depth investigation on the microstructural development during the processing of a similar alloy, Mg-0.7Al-0.3Ca-0.5Mn, was done recently by Liu et al. [73]. The evolution of second phase particles was analyzed after casting, extrusion, T4 and T6 heat treatments, thereby showing the distinctive changes from phases formed during casting C36 and C15 (and Al${}_{8}$Mn${}_{5}$) toward C14 and C15 (and β-Mn) after extrusion to the sole-hardening phase C15 (and β-Mn) in the T6 state. The highest strength values were reached in the as-extruded state, which also boasts the smallest grain size and the highest amount of hardening phases (in vol.%), the drawback being an elongation of <5%. The lower amount of precipitating C15 phases in T6 state is attributed to the formation and growth of β-Mn, which is able to bind large amounts of Al, accordingly reducing the available amount for the formation of Al${}_{2}$Ca phases.

Not only was extrusion used to shape the Mg–Al–Zn lean alloys, but rolled products were also investigated [24,58,74,75]. When analyzing sheet materials, it must be considered that these are mostly used for semi-finished products, which are subsequently further shaped in various sheet-forming processes. Therefore, the aim in sheet production is usually to achieve good formability of the sheets and the possible subsequent hardening processes of the shaped part. The deep-drawing behavior of sheets can be gauged, for example, with the Erichsen test, in which the achievable drawing depth in mm to the first visible crack is measured (I.E. value). It is well known that the intense texture occurring in Mg alloys is counterproductive for this type of forming; therefore, extensive efforts were made to reduce the texture intensity in Mg sheet materials [4].

As discussed by Sandlöbes et al. [24] the sharp texture and low forming capabilities of Mg sheets is caused by the predominant deformation by basal slip. To produce an alloy with increased activation of the pyramidal slip, a Mg-1.0Al-0.1Ca alloy was chosen, based on ab initio simulations. The Ca content can be fully dissolved during homogenization, and the forming of Ca-containing phases is avoided, producing a UTS of 220 MPa and a tensile elongation of 20% in the investigated sheets.

**Figure 7:**
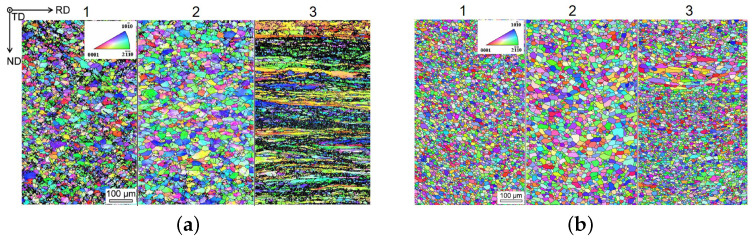
Evolution of microstructure and texture of a Mg-1.2Al-0.3Zn-0.3Ca-0.4Mn alloy, produced by rolling at (1) 100 ${}^{\circ }$C with reheating, (2) 300 ${}^{\circ }$C with reheating, (3) 300 ${}^{\circ }$C without reheating. Reheating took place at 500 ${}^{\circ }$C for 5 min before each rolling pass, and the solution heat treatment was done at 450 ${}^{\circ }$C for 1 h [75]. (**a**) As-rolled; (**b**) solution heat treated. Reprinted from Ref. [75] with permission; Copyright Elsevier 2018.

Chino et al. [58] studied the effect of Ca addition on the tensile properties and stretch formability of Mg–Al and Mg–Zn sheets. They reported good forming behavior due to texture softening and increased activation of prismatic slip. These findings were validated by Bian et al. [74], using Mg-1.2Al-0.5Ca-0.4Mn and Mg-1.2Al-0.8Zn-0.5Ca-0.4Mn alloys, which were investigated for their sheet forming and heat treatment behavior. In ref. [74] it was shown that the I.E. values of the AXMZ alloy (7.7 mm) were superior to the tested material without Zn (5.9 mm). The heat treatment performance and strength on the other hand were comparable for both types of alloys, reaching a YS of ~200 MPa and a UTS of ~260 MPa in the T6 state. The effects of varying rolling parameters on a similar alloy were analyzed in ref. [75], where low texture intensity and the activation of prismatic slip was found especially pronounced in a fine-grained homogeneous microstructure (see Figure 7), thereby reaching I.E. values of 7.0 mm. While the deformation behavior was strongly influenced by the processing parameters, the mechanical properties stayed constant in the T4 state.

### 4.2. Alloys Containing Zinc and Calcium

The main motivations for the development of Mg–Zn–Ca (ZX) alloys are their good forming behavior, their excellent biocompatibility and their adjustable degradation rate in body fluids, which is an important prerequisite for medical use as an implant material. Material strength, on the other hand, only plays a minor role. The good shaping characteristics of Mg–Zn alloys are also the reason for intensive investigation regarding their suitability for structural applications as extruded profiles or rolled sheets [4]. Sheets are often semi-finished products that are to be formed into their final shape in a concluding forming process, i.e., deep drawing, preferably at room temperature. Since the addition of Ca promotes the forming behavior, comparable to the addition of rare earths, dilute Mg–Zn–Ca is investigated in this respect as well.

The investigations of ZX alloys with low alloying content for bio-medical applications are focused on material showing optimal in-vivo degradation. Mg, Zn and Ca are essential trace elements in the human body, and ZX alloys can therefore be used for in vivo applications without medical incompatibility problems [19].

The interaction of Zn and Ca content was investigated by multiple authors [76,77,78], analyzing the possible precipitation hardening, grain growth and recrystallization behavior as well as deformation characteristics. A processing map calculated for an as-cast Mg-1.0Zn-1.0Ca alloy showed two favorable forming domains, featuring dynamic recrystallization, in a range of 280 to 330 ${}^{\circ }$C and a range of 330 to 400 ${}^{\circ }$C with possible forming speeds of the range 0.0003 to 0.01 s${}^{-1}$ and range 0.0003 to 0.1 s${}^{-1}$, respectively [76].

The precipitation hardening of cast and homogenized ZX alloys, with varying Zn content, were analyzed in ref. [77]. There, the best hardening results were achieved in a ZX21 alloy, where Zn and Ca containing G.P.- zones formed along the basal plane.

Interesting precipitation hardening results were also found in a hot rolled Mg-0.8Zn-0.2Ca alloy, where marginal plastic strain prior to an annealing treatment at a range of 80 to 200 ${}^{\circ }$C led to an increase in material strength [78]. In contrast, no precipitation strengthening effect could be found in annealed material without prior strain. It was shown that the basal dislocations produced by deformation were pinned by the annealing-induced formation of G.P.- zones, thereby improving the YS of the samples during repeated tensile testing.

Using such a Mg-0.8Zn-0.2Ca alloy, it was shown recently that the combination of Zn and Ca is necessary for a distinct improvement of room temperature ductility [59,79]. The material was analyzed after cold rolling and subsequent annealing [20] and hot rolling and annealing [80]. It was found that basal and pyramidal slip is increased at room temperature, and the twinning pushes toward higher strains. Subsequent annealing causes solute segregation and precipitation at the grain boundaries, promoting homogeneous grain size distributions by pinning. Nucleation of recrystallized grains starts preferably at tensile twins and shear bands. The combination of the described effects promotes the desired rare earth texture for these alloys.

The texture of a Mg-1.0Zn-0.9Ca alloy was shown to change noticeably during the processing steps in ref. [81]. While the texture from the hot rolled material showed a splitting of basal poles in the rolling direction, due to a strong formation of double twins, the subsequent annealing shifted the poles toward the transverse direction. In the analysis of the annealed sheets, an I.E. value of 8.8 mm and a UTS of 234.3 MPa was reached.

Investigations on the effect of Zn on Mg–Ca–Zr alloys showed a large increase in ductility and the potential for age hardening in a Mg-0.2Ca-0.4Zr-0.3Zn sheet [50]. The elongation to fracture was enhanced up to a value of 34% in TD because failure along the grain boundaries was suppressed. Additionally, this type of alloy can be age hardened, reaching a YS of 220 MPa in the T8 state, which accords to an increase of ~70 MPa. This was mostly achieved by the formation of nanoscale plate-like precipitates on the basal plane of the Mg matrix.

An increase in ductility was also found in ref. [82], where annealed sheets from Mg-1.2Zn-0.2Zr with and without Ca additions (0.4 wt.%) were compared. By Ca additions, a weak double peak texture was formed and the activation of {1012} twinning and pyramidal <c+a> slip was promoted. While the Ca containing sheets showed improved ductility and higher UTS, the mechanical anisotropy increased. In particular, the YS varied up to 50 MPa, depending on the testing direction, while the mechanical properties of the ZK10 alloy were more uniform overall [82].

A complementary study [83] analyzed the influence of Ca (0.4 and 0.8 wt.%) on an extruded and annealed Mg-1.4Zn-0.1MM-0.1Zr alloy. The Ca additions slightly reduced the overall material strength, with the YS being the most affected, decreasing by ~30 MPa to 170 MPa. Consequently, the yield anisotropy (YSc/YS) was shifted from 0.75 to 0.85. Solely, the alloy modified with 0.8 wt.% Ca showed microstructural changes, i.e., a reduced grain size, a higher degree of recrystallization and a reduction in texture intensity.

Mg-0.6Zn-0.6Ca-0.1Zr sheets, produced by twin-roll casting on an industrial scale, were investigated by Klaumünzer et al. [31] with regard to their forming behavior, using Erichsen testing. At RT, an I.E. value of ~7 mm was reached, and industrially relevant components could be formed at temperatures of 160 ${}^{\circ }$C. In ref. [32], this ZXK alloy was compared to Mg-1.8Zn-0.1Nd-0.1Ce-0.05La-0.2Y material, which showed comparable I.E. values at RT. The textures of both sheet materials are similar and show broad basal poles shifted toward the TD. The texture intensity depends on the rolling conditions but is weakened in the case of the annealed sheets. In general, the ZEW sheets featured higher strength and increased ductility compared to the ZXK alloy, which, on the other hand, exhibited reduced planar anisotropy. In the forming investigations (Erichsen testing and deep drawing of cups), the ZEW sheets showed better overall performance under all conditions and temperatures tested (up to 200 ${}^{\circ }$C), while the ZXK material failed due to crack initiation at the coarse MgCa particles [32].

As described in Section 4.1 extrusion is one of the preferred forming methods, which is why lean wrought Mg alloys are also intensively studied in this respect. An investigation of a Mg-1.0 Zn-0.5 Ca alloy illustrates the influence of the processing temperature on the mechanical properties of this material. In the experimental trials, the chosen extrusion temperature varied in the range of range of 310 to 400 ${}^{\circ }$C. A bimodal grain structure of small recrystallized and large deformed grains was formed below 350 ${}^{\circ }$C. This structure shows an increased yield strength but markedly reduced elongation to failure, when compared to samples formed at >350 ${}^{\circ }$C, which exhibit a fully recrystallized microstructure with increasing grain size at increasing extrusion temperatures [42].

In a following investigation, the effect of changing the Ca content (range 0 to 0.5 wt.%) on this alloy was analyzed [60]. The as-cast and subsequently extruded alloys displayed decreasing grain sizes and texture with raising the Ca content. It was shown that the rising amount of precipitates augmented the dynamic recrystallization but the subsequent grain growth was impeded. The resulting texture weakening caused a reduction in YS but a considerable increase in elongation to failure.

**Figure 8:**
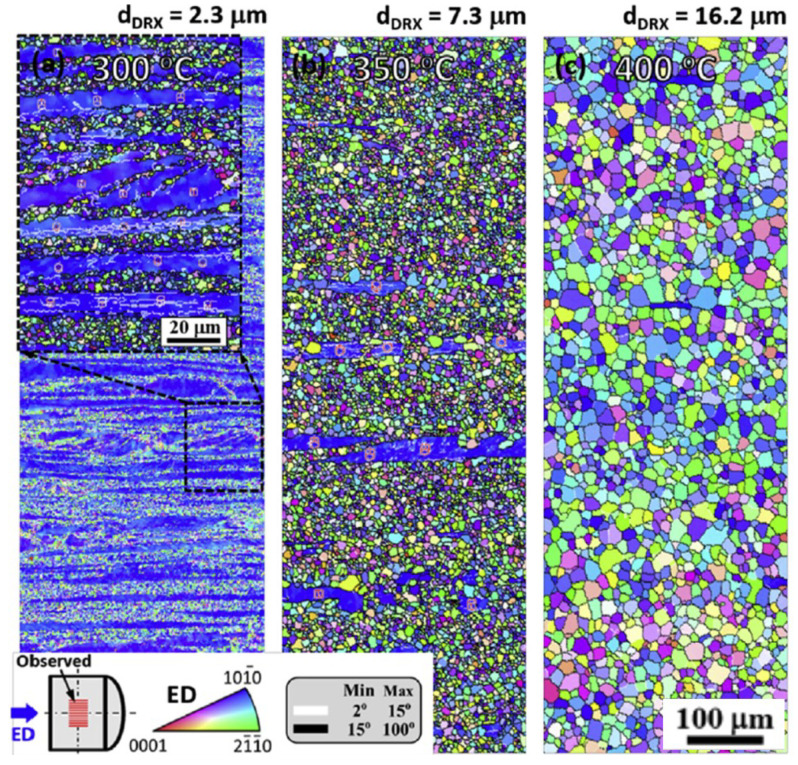
Microstructure and texture in an as-extruded Mg-0.2Zn-0.3Ca-0.1Mn alloy at rising processing temperatures: (**a**) 300 ${}^{\circ }$C, (**b**) 350 ${}^{\circ }$C, (**c**) 400 ${}^{\circ }$C [85]. Reprinted from Ref. [85] with permission; Copyright Elsevier 2016.

The positive effect of Ca-containing precipitations was confirmed in ref. [84], where dilute Mg–Zn–Ca–Mn alloys with increasing Zn content (0.2, 0.5 and 0.7 wt.%) were analyzed regarding their extrusion behavior. While all alloys could be formed with good die-exit speeds of 24 m min${}^{-1}$, only the alloy with the lowest Zn content (0.2 wt.%) could be extruded with die-exit speeds of up to 60 m min${}^{-1}$. The uniform elongation for all alloys and extrusion speeds was fairly consistent throughout, reaching ~23%. The tensile strength of the alloys on the other hand increased with rising Zn concentration and decreasing forming speed. This was attributed to the increase in solid solution strengthening by the Zn atoms, the lower forming temperature at low extrusion speeds and the resulting reduced grain size. It is assumed that the overall low yield strength (<110 MPa) of these alloys is due to the texture, which was found favorable for the activation of basal slip systems [84]. The microstructural behavior of the Mg-0.2Zn-0.3Ca-0.1Mn alloy was further analyzed in ref. [85], where the extrusion temperature was varied in the range of 300 to 400 ${}^{\circ }$C. The resulting microstructure changed from a bimodal structure of small recrystallized and larger deformed grains toward a fully recrystallized structure with rising forming temperatures; see Figure 8. All samples benefit from the precipitation of Mg${}_{2}$Ca and α-Mn phases, which inhibit grain growth. While the material processed at 300 ${}^{\circ }$C showed the best overall mechanical properties, with a YS of 307 MPa and an elongation of 20.6%, it also exhibited the strongest texture intensity and the strongest yield anisotropy (YS/YSc). The mechanical properties shifted to lower strength and higher elongation to failure for the samples produced with higher extrusion temperatures [85].

The applicability of lean alloyed ZX alloys for bio-medical applications, i.e., as biodegradable implant material, was the main motivation for the investigations made in refs. [19,86]. While the used elements Mg, Zn and Ca are essential trace elements and thus harmless for applications in the human body, the dissolution of these materials in vivo is a complex process. The degradation rate must be well controlled to avoid damage to the surrounding tissue and bone material by hydrogen production, which is a byproduct of the in vivo dissolution of Mg. Because of this, the possibilities to customize the mechanical properties by alloying are much reduced and the processing parameters, in addition to the absence of corrosion-promoting impurities, are the main means of adjustment [19].

As the mechanical properties inherently depend on the microstructural features generated during processing, the behavior of IMPs during extrusion is investigated by Hofstetter et al. [28]. There, the comparison of a Mg-0.5Zn-0.1Ca-0.1Mn alloy (without IMPs) and Mg-1.0Zn-0.3Ca (with IMPs) illustrates the large influence of the dispersed Mg${}_{2}$Ca precipitates on grain size (<2 μm) and tensile properties. Consequently, the Mg-1.0Zn-0.3Ca alloy achieved a YS of 238 MPa and a UTS of 265 MPa with an elongation to fracture of 31%.

A different approach was used in ref. [41] where SPD was used to improve the mechanical properties of a homogenized Mg-0.8Zn-0.2Ca alloy. By a subsequent use of ECAP and HPT, a UTS of up to 283 MPa was reached.

To adjust the biodegradability to an optimum level, in depth studies on corrosion and the corresponding microstructural features were performed.

Cihova et al. [86] stated that the Zn content of ZX alloys has a crucial impact on the corrosion behavior. This is caused by an enhancement of the cathodic activity through Zn re-deposition and the forming of various IMPs, Mg${}_{2}$Ca or Ca${}_{2}$Mg${}_{6}$Zn${}_{3}$, which is a controlling feature of the degradation behavior of these alloys. The Ca${}_{2}$Mg${}_{6}$Zn${}_{3}$ phases act as cathodes and promote the Mg matrix degradation, while the Mg${}_{2}$Ca phases dissolve before the matrix, resulting in a slow and homogeneous degradation of the material. The forming of the Mg${}_{2}$Ca phase is, therefore, one of the main factors for the reduced corrosion behavior of the tested Mg-1.0Zn-0.3Ca lean alloy, which was shown to be well suitable for in vivo applications [86].

**Figure 9:**
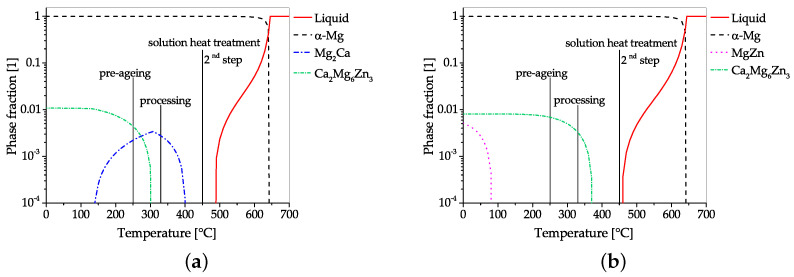
CALPHAD calculations, including the used processing temperatures, of two biodegradable Mg–Zn–Ca alloys. Calculations were made, using the alloy compositions given in ref. [86]; for further information see Appendix A.2. (**a**) Mg-1.0Zn-0.3Ca; (**b**) Mg-1.5Zn-0.2Ca.

The alloying and processing scheme used in ref. [86] is depicted in the CALPHAD calculations given in Figure 9. This is an interesting example of how small changes in the alloying content can have a distinctive influence on the material properties (discussed above), as processing is the same for both alloys.

The alloy Mg-1.0Zn-0.3Ca allows for the formation of both the Ca${}_{2}$Mg${}_{6}$Zn${}_{3}$ and Mg${}_{2}$Ca phases, while only the Ca${}_{2}$Mg${}_{6}$Zn${}_{3}$ phase may precipitate in the Mg-1.5Zn-0.2Ca alloy. The homogenization temperature (up to 450 ${}^{\circ }$C) was chosen to be as high as possible, restricted by the Mg-1.5Zn-0.2Ca alloy, which has the lower solidus temperature. IMPs, which could have formed during casting, are fully soluble in both alloys, thereby producing a super saturated solid solution during the homogenization heat treatment [86]. To avoid grain growth during extrusion, the alloys are pre-aged, forming Ca-containing precipitates, as described in ref. [28]. The CALPHAD calculations show that a precipitation of both the Ca${}_{2}$Mg${}_{6}$Zn${}_{3}$ and Mg${}_{2}$Ca phases is possible in the Mg-1.0Zn-0.3Ca alloy during pre-aging. Nevertheless, only the Mg${}_{2}$Ca phase is present during and after the extrusion process. The Mg-1.5Zn-0.2Ca alloy, on the other hand, retains the ternary phase throughout extrusion. This differing precipitation behavior is, as already discussed above, the controlling feature for the corrosion behavior of these alloys. While, based on the thermodynamic calculations, it may be assumed that dynamic precipitation of the respective IMPs occurs during processing, this has not been discussed in the cited literature [28,86].

### 4.3. Alloys Containing Various Alloying Elements

This section discusses publications using various alloying schemes not specifically mentioned in the previous sections, which were accordingly restricted to lean alloys containing Al–Ca and Zn–Ca. Therefore, this section contains a broader variation of alloy compositions, i.e., Mg–RE and Mg–Sn alloys.

The **combination of Zn and REE** (sometimes refined by **Zr additions**) is an often-chosen alloying route, known to produce alloys with good forming performances. Some of the works [32,83] investigating these alloys were already mentioned in Section 4.2.

The influence of micro-alloying with Ge on the corrosion behavior of a cast and extruded lean Mg–Zn alloys in saline solution was investigated by Jiang et al. [87,88]. As the investigated Mg-0.5Zn-0.2Ge alloy featured Mg${}_{2}$Ge phases, which are nobler than the surrounding Mg matrix, localized corrosion of the matrix was found in the cast alloy. This behavior changed in the as-extruded material where uniform corrosion was prevalent. In a comparison with a Mg-0.5Zn-0.2Ca alloy, the Ge containing material showed superior tensile strength and improved corrosion behavior. This slightly higher corrosion resistance of the Mg-0.5Zn-0.2Ge alloy is due to an increased incorporation of Zn and Ge in the outer layer of the corrosion products film and was less affected by corrosion in short term fatigue tests.

The investigations made by Al-Samman and Li [89] analyzed the effect of different REE additions (Gd, Nd, Ce, La and mischmetal (MM)) on Mg–Zn–Zr alloys, giving insight into the rolling and annealing behavior. All of the RE elements investigated led to a weakening of the rolling texture, which was further reduced by an annealing treatment. This typical behavior for REE containing alloys was confirmed by the tensile properties, showing an overall reduction in mechanical anisotropy and high ductility (up to 32% true strain). The biggest effect was found in the Gd-containing alloy, reflected by the strong texture modification and superior ductility at room temperature, despite exhibiting the coarsest grain structure compared to the other alloys [89].

A Mg-1.6Zn-0.5Gd alloy, analyzed with regard to the processing performance [43,90], microstructural features and recrystallization behavior [91] showed favorable forming surfaces, even when produced by high extrusion rates, up to 60 m min${}^{-1}$ die-exit speed. The tensile properties, however, changed considerably with the processing conditions, e.g., the extrusion rate and temperature or pre-deformation. Nevertheless, high values for elongation to failure were measured throughout [90]. It could be shown that the precipitation of Mg${}_{3}$Zn${}_{3}$Gd${}_{2}$ particles during the extrusion hinders grain growth, by forming at the grain boundaries and the grain interior [43]. While the texture of the dynamically recrystallized microstructure features [2110] basal fiber components, the typical REE texture mainly develops during the subsequent static recrystallization [91].

The influence of extrusion speed on microstructure and mechanical properties in combination with alloy modifications is an often investigated topic, as it is an essential topic for industrial applications. The effect of Zr additions (0.5 wt.%) on a Mg-1.3Zn-0.1Ce alloy was studied in ref. [92], comparing indirect extrusions with die-exit speeds of 1, 10 and 20 m min${}^{-1}$. With a rising extrusion speed, the amount of recrystallized grains increases for both alloys, while the tensile and compressive strengths decrease markedly. Elongations to break, on the other hand, are less dependent on the extrusion rate. The modified Mg-1.3Zn-0.2Ce-0.5Zr alloy exhibits a smaller grain size and higher strength values throughout all processing speeds. Interestingly, the grain size does not change with the increase in the processing speed to 20 m min${}^{-1}$, but stays constant at ~7 μm in this alloy [92].

Complementary investigations were made on ZK alloys, where a Mg-1.0Zn-0.4Zr lean alloy was modified with 0.8 wt.% Ce rich MM [93,94]. Extrusion trials took place with die-exit speeds of 1, 5 and 10 m min${}^{-1}$, showing increased grain size, decreasing YS and UTS as well as a reduced overall texture intensity with the rising processing speeds. A smaller grain size and higher strength was found in the MM-containing alloy.

Mg alloys forming long period stacking order (LPSO) phases show interesting mechanical properties, e.g., high temperature strength. The desired properties of these alloys can be directly correlated to the volume fraction of the LPSO phase, which increases with the rising alloying content, as reported in ref. [95]. There, a Mg-0.5Zn-2.2Y alloy was found to reach only 1% of the LPSO volume fraction, showing accordingly reduced properties when compared to the alloys with a higher element content. As the possible amount of formed LPSO phase fraction is also controlled by the used Zn/Y ratio, optimization in this regard can lead to increased LPSO phase fractions at comparable overall alloying contents [96]. Nevertheless, it seems questionable whether this principle of ordered Mg–Zn–Y(/Gd) phases can a show a feasible effect in lean alloys as well.

The influence of precipitation formation and recrystallization is also given special attention for alloys of the **Mg–Mn–RE system**.

In ref. [97], a Mg-1.8Mn alloy was modified with Er and Er/Al additions, homogenized and extruded. The subsequently annealed samples showed increasing elongation to break with rising alloying additions. In the case of the Er containing alloys, the fraction of recrystallized grains varied between 45 and 100%. The alloys modified with a combination of Er and Al featured an increasing recrystallized fraction (range 60 to 80%) with the amount of alloying elements, also showing a superior elongation to break during tensile testing.

Extensive investigations on an extruded Mg-1.0Mn-1.0Nd alloy were made to analyze the precipitation behavior and the reversed yield asymmetry (YSc > YS), which appears at various temperatures [98]. During annealing at 250 ${}^{\circ }$C, the precipitating Mg${}_{3}$Nd−plates orient themselves along the c-axis, causing anisotropic strengthening effects. This causes a preferred deformation via prismatic slip, promoting the yield strength asymmetry at RT. On the contrary, the reason for this effect at high temperatures is thought to be caused by the mobility of the pyramidal <c+a> dislocations [99].

Investigations on **Mg-Sn lean alloys** indicate good RT forming behavior of extruded sheet material. In a comparison of multiple alloys, both Mg-1.3Sn-0.7Ca and Mg-1.1Sn-0.6Zn-0.5Ca exhibited split basal textures after extrusion [100]. The results of tensile testing in TD indicate a better performance of the Mg-1.1Sn-0.6Zn-0.5Ca alloy. This is attributed to increased activation of the prismatic slip, enhanced grain boundary cohesion and improved intergranular strain propagation capacity.

The addition of Y to lean Mg–Sn alloys influences the mechanical properties of as-extruded sheets at RT positively, especially the elongation to break showed marked improvement. Investigations on Mg-0.3Sn-0.7Y [101] and Mg-0.5Sn-0.3Mn-0.3Y [102] alloys attributed this to a decreased grain size, favorable texture and the increased activity of non-basal slip when compared to the alloys without Y. Subsequent work confirmed this also for rolled and annealed sheets made from Mg-0.3Sn-0.7Y [103]. Further improvements of the mechanical properties were achieved with a dilute addition of Zn, producing a Mg-0.4Sn-0.6Zn-0.7Y alloy with decreased grain and particle size, increasing the I.E. value from a range of 6.2 to 7.0 mm [104].

## 5. Concluding Remarks

This review is intended to provide an overview of the works published on Mg alloys with multiple alloying elements, up to a sum of ~2 wt.% and ~1 at.% of additions. The provided excerpt of the current state of research may serve as an introduction to the various possibilities in the diverse family of Mg lean alloys.

The investigations of these so-called low, dilute, HSLA or lean alloys offer a broad variety of properties, processes and applications, ranging from age hardened alloys with very high processing rates to biomedical applications with controllable in vivo dissolution.

While most of the works discussed highlight the practical applications and the improvements made by this alloying concept, there are also some drawbacks that need to be addressed. Mechanical properties, which rely on an increased amount of alloying elements, such as the high contribution of solid solution hardening, the LPSO effect, or generally those where the focus lies on high strength values, cannot fully be realized with the lean alloying concept. The discussed alloys often operate in a narrow range or make use of (thermodynamic) features that are strongly dependent on exact chemical compositions. Some may also show a unusually high response to impurities. These characteristics and the sometimes sensitive casting behavior increases the difficulty for stock material production and may impede recyclability.

Regardless of the limitations mentioned above, the ’lean alloy’ design concept opens up a wide range of opportunities for the further development of Mg wrought alloys and is strongly orientated toward suitability for everyday use. The reduction and focus on easily available alloying elements, the full utilization of mechanical properties, i.e., improved ductility or age hardening, increase the viability of these alloys markedly. Therefore, demands of industrial manufacturing, such as high extrusion speed or complex secondary sheet forming operations, can be met easier. The development of these materials is not yet complete, but the successes so far promise an exciting field of work as well as multiple applications in the near future.

The authors hope that this brief introduction to the diverse topic of ’Lean Wrought Magnesium Alloys’ can be a stimulus for further development and an aid to the visibility of these interesting alloys.

## Abbreviations

The following abbreviations and symbols are used in this manuscript:

CRSS	critical resolved shear stress	SPD	severe plastic deformation
ECAP	equal channel angular pressing	UCS	ultimate compressive strength
G.P.- zone	Guinier-Preston zone	UTS	ultimate tensile strength
HPT	high pressure torsion	YS	tensile yield strength
HSLA	High Strength Low Alloyed	YSc	compressive yield strength
I.E.-value	Erichsen Index		
IMP	intermetallic particle/phase	$\epsilon_{f}$	elongation to failure
LPSO	long period stacking order	*d*	grain diameter
MAF	multi axial forging	k${}_{y}$	Hall Petch slope
MM	mischmetal	$\sigma_{0}$	frictional stress
RE/REE	rare earth elements	at.%	atomic percent
RT	room temperature	wt.%	weight percent

## Appendix A. Additional Information

### Appendix A.1. Sources for Literature Research

The literature research for this work was primarily done online, where the following online sources were mainly used: Scopus, Google Scholar, Web of Science and Espacenet.

### Appendix A.2. CALPHAD Calculations

The figures showing CALPHAD calculations were done with the software Thermo-Calc Version 2021a and the database Thermo-Calc TCMG5. The alloy compositions used are listed in Table A1.

## Appendix B. Alloy Overview

This section lists the chemical compositions (in wt.% and at.%) of the investigated Mg alloys. As the discussed works often analyze multiple alloys with varying alloy content, Table A1 also includes materials slightly outside of the designated range of 1 at.% and 2 wt.%, where they are thought appropriate and useful for comparison.

**Table A1:** Chemical composition of alloys discussed in the reviewed works, given in  wt.% and (at.%). Alloying content below 0.01% (100 ppm) is not listed; the RE elements are combined into a single column for improved readability.

	Alloy	Source	∑ Elements	Mg	Al	Zn	Ca	Mn	Si	∑ REE	Sn	Zr
**Mg-Al-Ca**	Mg-0.1Al-0.5Ca	[26,48]	0.60 (0.39)	Bal	0.10 (0.09)		0.50 (0.30)					
	Mg-0.3Al-0.2Ca	[26,48]	0.50 (0.39)	Bal	0.30 (0.27)		0.20 (0.12)					
	Mg-0.3Al-0.2Ca-0.5Mn	[9,40]	0.98 (0.61)	Bal	0.30 (0.27)		0.21 (0.13)	0.47 (0.21)				
	Mg-0.3Al-0.3Ca	[70]	0.64 (0.48)	Bal	0.32 (0.29)		0.32 (0.19)					
	Mg-0.3Al-0.3Ca-0.2Mn	[70]	0.91 (0.61)	Bal	0.33 (0.30)		0.34 (0.20)	0.24 (0.11)				
	Mg-0.3Al-0.3Ca-0.4Mn	[70,71]	1.09 (0.69)	Bal	0.34 (0.31)		0.32 (0.19)	0.43 (0.19)				
	Mg-0.3Al-0.3Ca-0.8Mn	[70]	1.45 (0.84)	Bal	0.30 (0.27)		0.31 (0.19)	0.84 (0.37)				
	Mg-0.6Al-0.3Ca-0.3Mn	[23,72]	1.13 (0.82)	Bal	0.60 (0.54)		0.28 (0.17)	0.25 (0.11)				
	Mg-0.6Al-0.5Ca	[10]	1.07 (0.83)	Bal	0.61 (0.55)		0.46 (0.28)					
	Mg-0.7Al-0.3Ca-0.5Mn	[73]	1.50 (1.04)	Bal	0.68 (0.62)		0.32 (0.20)	0.50 (0.22)				
	Mg-1.0Al-0.1Ca	[24]	1.10 (0.96)	Bal	1.00 (0.90)		0.10 (0.06)					
	Mg-1.0Al-1.0Zn-0.1Ca-0.5Mn	[58]	2.60 (1.57)	Bal	1.00 (0.91)	1.00 (0.38)	0.10 (0.06)	0.50 (0.22)				
	Mg-1.2Al-0.3Zn-0.3Ca-0.4Mn	[75]	2.20 (1.60)	Bal	1.20 (1.10)	0.30 (0.10)	0.30 (0.20)	0.40 (0.20)				
	Mg-1.2Al-0.5Ca-0.4Mn	[74]	2.10 (1.60)	Bal	1.20 (1.10)		0.50 (0.30)	0.40 (0.20)				
	Mg-1.2Al-0.8Zn-0.5Ca-0.4Mn	[74]	2.90 (1.90)	Bal	1.20 (1.10)	0.80 (0.30)	0.50 (0.30)	0.40 (0.20)				
	Mg-1.3Al-0.3Ca-0.5Mn	[71]	2.10 (1.60)	Bal	1.31 (1.19)		0.33 (0.20)	0.46 (0.21)				
	Mg-2.7Al-0.3Ca-0.4Mn	[71]	3.51 (2.88)	Bal	2.73 (2.48)		0.34 (0.20)	0.44 (0.20)				
**Mg-Zn-Ca**	Mg-0.2Zn-0.3Ca-0.1Mn	[84,85]	0.65 (0.32)	Bal		0.21 (0.08)	0.30 (0.18)	0.14 (0.06)				
	Mg-0.3Zn-0.5Ca	[77]	0.80 (0.40)	Bal		0.30 (0.10)	0.50 (0.30)					
	Mg-0.8Ca-0.37Zr	[50]	1.19 (0.60)	Bal			0.82 (0.50)					0.37 (0.10)
	Mg-0.8Ca-0.37Zr-0.3Zn	[50]	1.46 (0.70)	Bal		0.27 (0.10)	0.82 (0.50)					0.37 (0.10)
	Mg-0.5Zn-0.1Ca-0.1Mn	[28]	0.69 (0.31)	Bal	0.02 (0.02)	0.48 (0.18)	0.14 (0.09)	0.03 (0.01)	0.02 (0.02)			
	Mg-0.5Zn-0.2Ca-0.3Mn	[84]	1.04 (0.46)	Bal		0.53 (0.20)	0.24 (0.15)	0.27 (0.12)				
	Mg-0.6Zn-0.6Ca-0.1Zr	[31,32]	1.30 (0.62)	Bal		0.60 (0.22)	0.60 (0.37)					0.10 (0.03)
	Mg-0.7Zn-0.4Ca-0.1Mn	[84]	1.14 (0.52)	Bal		0.71 (0.27)	0.36 (0.22)	0.07 (0.03)				
	Mg-0.8Zn-0.2Ca	[59]	1.02 (0.43)	Bal		0.80 (0.30)	0.20 (0.12)	0.02 (0.01)				
	Mg-0.8Zn-0.2Ca	[20,78,79,80]	0.96 (0.40)	Bal		0.80 (0.30)	0.16 (0.10)					
	Mg-0.8Zn-0.5Ca	[77]	1.30 (0.60)	Bal		0.80 (0.30)	0.50 (0.30)					
	Mg-1.0Zn-0.2Ca	[41,60]	1.20 (0.49)	Bal		1.00 (0.37)	0.20 (0.12)					
	Mg-1.0Zn-0.3Ca	[19]	1.30 (0.56)	Bal		1.00 (0.37)	0.30 (0.18)					
	Mg-1.0Zn-0.3Ca	[28]	1.33 (0.59)	Bal	0.02 (0.02)	0.96 (0.36)	0.29 (0.18)	0.03 (0.01)	0.03 (0.03)			
	Mg-1.0Zn-0.3Ca	[86]	1.28 (0.56)	Bal		0.96 (0.36)	0.32 (0.20)					
	Mg-1.0Zn-0.5Ca	[42,60]	1.50 (0.68)	Bal		1.00 (0.37)	0.50 (0.31)					
	Mg-1.0Zn-0.9Ca	[81]	1.85 (0.91)	Bal		0.95 (0.36)	0.90 (0.55)					
	Mg-1.2Zn-0.4Ca-0.2Zr	[82]	1.78 (0.74)	Bal		1.21 (0.45)	0.39 (0.24)					0.18 (0.05)
	Mg-1.4Zn-0.1MM-0.1Zr-0.4Ca	[83]	2.00 (0.83)	Bal		1.40 (0.53)	0.40 (0.25)			0.10 (0.02)		0.10 (0.03)
	Mg-1.4Zn-0.1MM-0.1Zr-0.8Ca	[83]	2.40 (1.07)	Bal		1.40 (0.53)	0.80 (0.49)			0.10 (0.02)		0.10 (0.03)
	Mg-1.5Zn-0.1Ca	[58]	1.60 (0.62)	Bal		1.50 (0.56)	0.10 (0.06)					
	Mg-1.5Zn-0.2Ca	[86]	1.72 (0.71)	Bal		1.48 (0.56)	0.24 (0.15)					
	Mg-1.6Zn-0.5Ca	[77]	2.10 (0.90)	Bal		1.60 (0.60)	0.50 (0.30)					
	Mg-2.6Zn-0.5Ca	[77]	3.10 (1.30)	Bal		2.60 (1.00)	0.50 (0.30)					
**Various**	Mg-1.0Mn-1.0Nd	[98,99]	2.00 (0.62)	Bal				1.00 (0.45)		1.00 (0.17)		
	Mg-1.8Mn-0.1Er	[97]	1.90 (0.82)	Bal				1.80 (0.81)		0.10 (0.01)		
	Mg-1.8Mn-0.1Er-0.1Al	[97]	1.95 (0.87)	Bal	0.05 (0.05)			1.80 (0.81)		0.10 (0.01)		
	Mg-1.8Mn-0.4Er	[97]	2.20 (0.87)	Bal				1.80 (0.81)		0.40 (0.06)		
	Mg-1.8Mn-0.4Er-0.2Al	[97]	2.40 (1.05)	Bal	0.20 (0.18)			1.80 (0.81)		0.40 (0.06)		
	Mg-1.8Mn-0.7Er	[97]	2.50 (0.91)	Bal				1.80 (0.81)		0.70 (0.10)		
	Mg-1.8Mn-0.7Er-0.3Al	[97]	2.84 (1.22)	Bal	0.34 (0.31)			1.80 (0.81)		0.70 (0.10)		
	Mg-0.3Sn-0.7Y	[101,103,104]	1.04 (0.27)	Bal						0.71 (0.20)	0.33 (0.07)	
	Mg-0.4Sn-0.7Y-0.6Zn	[104]	1.70 (0.51)	Bal		0.64 (0.24)				0.65 (0.18)	0.41 (0.09)	
	Mg-0.5Sn-0.3Mn	[102]	0.82 (0.23)	Bal				0.27 (0.12)			0.55 (0.11)	
	Mg-0.5Sn-0.3Mn-0.3Y	[102]	1.08 (0.31)	Bal				0.27 (0.12)		0.33 (0.09)	0.48 (0.10)	
	Mg-1.1Sn-0.6Zn-0.5Ca	[100]	2.12 (0.73)	Bal		0.55 (0.21)	0.47 (0.29)				1.10 (0.23)	
	Mg-1.2Sn-0.5Zn	[100]	1.64 (0.42)	Bal		0.48 (0.18)					1.16 (0.24)	
	Mg-1.3Sn-0.7Ca	[100]	1.97 (0.70)	Bal			0.69 (0.43)				1.28 (0.27)	
	Mg-0.5Zn-0.2Ca	[88]	0.74 (0.34)	Bal		0.47 (0.18)	0.23 (0.14)	0.03 (0.01)	0.01 (0.01)			
	Mg-0.5Zn-0.2Ge	[87,88]	0.73 (0.27)	Bal		0.49 (0.18)		0.03 (0.01)	0.01 (0.01)	0.20 (0.07)		
	Mg-0.5Zn-2.2Y	[95]	2.68 (0.80)	Bal		0.53 (0.20)				2.15 (0.60)		
	Mg-0.6Zn-0.3Zr-0.6Nd	[89]	1.46 (0.40)	Bal		0.61 (0.23)				0.58 (0.10)		0.27 (0.07)
	Mg-0.7Zn-0.2Zr-0.7Gd	[89]	1.70 (0.44)	Bal		0.73 (0.27)				0.73 (0.11)		0.24 (0.06)
	Mg-0.7Zn-0.2Zr-0.8Ce	[89]	1.64 (0.45)	Bal		0.68 (0.26)				0.78 (0.14)		0.18 (0.05)
	Mg-0.8Zn-0.3Zr-0.9MM	[89]	2.05 (0.57)	Bal		0.84 (0.32)				0.88 (0.16)		0.33 (0.09)
	Mg-0.9Zn-0.2Zr-0.7La	[89]	1.80 (0.52)	Bal		0.89 (0.34)				0.69 (0.12)		0.22 (0.06)
	Mg-1.0Zn-0.4Zr	[94]	1.40 (0.49)	Bal		1.00 (0.38)						0.40 (0.11)
	Mg-1.0Zn-0.4Zr-0.8MM	[94]	2.20 (0.63)	Bal		1.00 (0.38)				0.80 (0.14)		0.40 (0.11)
	Mg-1.2Zn-0.2Zr	[82]	1.39 (0.51)	Bal		1.22 (0.46)						0.17 (0.05)
	Mg-1.3Zn-0.1Ce	[92]	1.40 (0.51)	Bal		1.30 (0.49)				0.10 (0.02)		
	Mg-1.3Zn-0.2Ce-0.5Zr	[92]	2.00 (0.67)	Bal		1.30 (0.49)				0.20 (0.04)		0.50 (0.14)
	Mg-1.4Zn-0.1MM-0.1Zr	[83]	1.50 (0.58)	Bal		1.40 (0.53)				0.10 (0.02)		
	Mg-1.6Zn-0.5Gd	[43,90,91]	2.10 (0.68)	Bal		1.58 (0.60)				0.52 (0.08)		
	Mg-1.8Zn-0.1Nd-0.1Ce-0.05La-0.2Y	[32]	2.26 (0.79)	Bal		1.81 (0.68)				0.45 (0.11)		

## Appendix C. Mechanical Properties

This section aims to give an overview of the mechanical properties of the discussed Mg alloys. For clarification, the temper designations used throughout this work are listed in Table A2.

Additional information on the Hall–Petch parameters depicted in Figure 3 is compiled in Table A3.

Table A4 summarizes the mechanical properties of the selected materials, discussed in Appendix B at RT. The values are directly taken from the works cited and may include stock material. The respective processing parameters are listed as thoroughly as feasible.

**Table A2:** Temper designations used in this work.

Designation	Explanation
F	as-fabricated
O	annealed, recrystallized
T4	solution heat treated and naturally aged ${}^{‡}$
T5	artificially aged only
T6	solution heat treated and artificially aged
T8	solution heat treated, cold worked and artificially aged

^‡^: As the effect of natural aging in the described Mg alloys is neglectable, solution heat treated material can also be referred to as T4 temper.

**Table A3:** Overview of the alloys and corresponding constants, Hall–Petch slope (k${}_{y}$) and frictional stress ($\sigma_{0}$) used in Figure 3. The Hall–Petch relationship $YS=k_{y}*d^{-1/2}+\sigma_{0}$, where *d* represents the grain diameter, was used for the calculation of the YS depicted in Figure 3b. The values of the alloys marked with an asterisk (*) have been calculated from the microstructure given in the corresponding source literature.

Alloy	k${}_{y}$ [MPa μm^2^]	$\sigma_{0}$ [MPa]	Source
Mg-0.3Al-0.2Ca-0.5Mn	208	143	[40]
Mg-1.0Zn-0.2Ca *	72	121	[41]
Mg-1.0Zn-0.3Ca	241	47	[28]
Mg-1.0Zn-0.5Ca *	236	37	[42]
Mg-1.6Zn-0.5Gd *	411	45	[43]

**Table A4:** Overview of the mechanical properties (at RT) and processing parameters of the alloys discussed in this work. The following abbreviations are exclusively used in this table: extrusion (extr.), homogenization (hom.), temperature (temp.), material (mat.), solution heat treatment (s.h.t.), water quenched (w.q.), without (w/o), subsequently (subseq.).

	Alloy	Temper	YS [MPa]	UTS [MPa]	$\epsilon_{f}$ [%]	YSc [MPa]	UCS [MPa]	Source		Process Parameters
**Mg-Al-Ca**	Mg-0.3Al-0.2Ca-0.5Mn	as-extr.	170		15.5			[9]		cast, hom. at 500 ${}^{\circ }$C for 24 h, w.q., extr. to round bars (indirect, 1:20) at 500 ${}^{\circ }$C with a die-exit speed of 60 m min${}^{-1}$
		T5	207		12.5			[9]		as above, aged at 200 ${}^{\circ }$C for 7 h
		as-extr.	206		29			[40]		cast, extr. to round bars (indirect, 1:20) at 350 ${}^{\circ }$C with a die-exit speed of 60 m min${}^{-1}$
		as-extr.	177					[40]		cast, extr. to round bars (indirect, 1:20) at 500 ${}^{\circ }$C with a die-exit speed of 60 m min${}^{-1}$
	Mg-0.3Al-0.3Ca-0.2Mn	as-extr.	136 ± 2	203 ± 2	29 ± 2			[70]		cast, hom. at 450 ${}^{\circ }$C for 1 h, w.q., extr. to round bars (indirect w/o lubricant, 1:20) at 400 ${}^{\circ }$C with a die-exit speed of 60 m min${}^{-1}$
		T6	188±0	247±0	27±0			[70]		as above, s.h.t. (500 ${}^{\circ }$C for 10 min) and w.q., aged at 200 ${}^{\circ }$C
	Mg-0.3Al-0.3Ca-0.4Mn	as-extr.	166 ± 1	220 ± 1	24±0			[70]		cast, hom. at 450 ${}^{\circ }$C for 1 h, w.q., extr. to round bars (indirect w/o lubricant, 1:20) at 400 ${}^{\circ }$C with a die-exit speed of 60 m min${}^{-1}$
		T6	220 ± 2	264±0	22 ± 2			[70]		as above, s.h.t. (500 ${}^{\circ }$C for 10 min) and w.q., aged at 200 ${}^{\circ }$C
		T4/s.h.t.	159 ± 3	228 ± 1	29 ± 1	119 ± 5	405 ± 4	[71]		cast, hom. at 500 ${}^{\circ }$C for 1 h, extr. to round bars (indirect, 1:20) at 275 ${}^{\circ }$C with a die-exit speed of 24 m min${}^{-1}$, s.h.t. (500 ${}^{\circ }$C for 10 min) and w.q.
		T5	230 ± 5	262 ± 3	26 ± 1	165 ± 2	434 ± 1	[71]		as above, aged at 200 ${}^{\circ }$C for 8 h
	Mg-0.3Al-0.3Ca-0.8Mn	as-extr.	190 ± 3	229 ± 1	24 ± 3			[70]		cast, hom. at 450 ${}^{\circ }$C for 1 h, w.q., extr. to round bars (indirect w/o lubricant, 1:20) at 400 ${}^{\circ }$C with a die-exit speed of 60 m min${}^{-1}$
		T6	199 ± 1	243±0	22 ± 3			[70]		as above, s.h.t. (500 ${}^{\circ }$C for 10 min) and w.q., aged at 200 ${}^{\circ }$C
	Mg-0.6Al-0.3Ca-0.3Mn	as-extr.	165 ± 7.8	232 ± 2.7	14 ± 0.3			[23,72]		cast, hom. at 480 ${}^{\circ }$C for 6 h, w.q., extr. to round bars (direct, 1:25) at 450 ${}^{\circ }$C with a die-exit speed of range 0.75 to 3.75 m min${}^{-1}$
		T5	236 ± 2.1	265 ± 1.9	7 ± 11.0			[23,72]		as above, aged at 200 ${}^{\circ }$C
		T4/s.h.t.	156 ± 0.1	225 ± 5.3	9 ± 2.5			[23,72]		extruded as above, s.h.t. (510 ${}^{\circ }$C for 10 min) and w.q.
		T6	253 ± 0.2	277 ± 4.2	8 ± 2.9			[23,72]		as above, aged at 200 ${}^{\circ }$C
	Mg-0.6Al-0.5Ca	as-extr.	182	250	19.4			[10]		cast, hom. at 400 ${}^{\circ }$C for 24 h, extr. (direct, 1:28) at 400 ${}^{\circ }$C with speed of 2 mm s${}^{-1}$
	Mg-0.7Al-0.3Ca-0.5Mn	as-cast	35 ± 1.0	106 ± 1.2	5.9 ± 0.1			[73]		cast in steel mold
		as-extr.	316 ± 6.0	327 ± 2.3	4.3 ± 0.5			[73]		cast, hom. at 500 ${}^{\circ }$C for 4 h, extr. (1:12) at 350 ${}^{\circ }$C with ram-speed of 0.1 mm s${}^{-1}$
		T4	162 ± 1.2	247 ± 1.9	25.4 ± 0.8			[73]		as above, s.h.t. (500 ${}^{\circ }$C for 10 min)
		T6	248 ± 1.5	288 ± 3.0	21.0 ± 0.4			[73]		as above, aged at 200 ${}^{\circ }$C for 16 h
	Mg-1.0Al-0.1Ca	rolled, O, TD		220	~20			[24]		cast, hot rolled at 430 ${}^{\circ }$C to SI50% thickness, annealed at 450 ${}^{\circ }$C for 0.25 h, w.q.
	Mg-1.2Al-0.3Zn-0.3Ca-0.4Mn	T4/s.h.t.	~125	~220	~30			[75]		cast, hom. at 300 ${}^{\circ }$C for 4 h followed by 500 ${}^{\circ }$C for 6 h and w.q., rolling at various conditions, s.h.t. at 450 ${}^{\circ }$C for 1 h
	Mg-1.2Al-0.5Ca-0.4Mn	T4	145	229	28			[74]		cast, hom. at 300 ${}^{\circ }$C for 4 h followed by 450 ${}^{\circ }$C for 6 h and w.q., rolling at 300 ${}^{\circ }$C (from range 10 to 5 mm, 4 passes), subsequent rolling at 100 ${}^{\circ }$C (from range 5 to 1 mm, 6 passes, reheated to 450 ${}^{\circ }$C for 5 min, solutionized at 450 ${}^{\circ }$C for 1 h
		T6	196	256	27			[74]		as above, aged at 200 ${}^{\circ }$C for 1 h
	Mg-1.2Al-0.8Zn-0.5Ca-0.4Mn	T4	144	242	32			[74]		cast, hom. at 300 ${}^{\circ }$C for 4 h followed by 450 ${}^{\circ }$C for 6 h and w.q., rolling at 300 ${}^{\circ }$C (from range 10 to 5 mm, 4 passes), subsequent rolling at 100 ${}^{\circ }$C (from range 5 to 1 mm, 6 passes, reheated to 450 ${}^{\circ }$C for 5 min, solutionized at 450 ${}^{\circ }$C for 1 h
		T6	204	263	27			[74]		as above, aged at 200 ${}^{\circ }$C for 1 h
	Mg-1.3Al-0.3Ca-0.5Mn	T4/s.h.t.	187 ± 1	267 ± 2	18 ± 1	138 ± 3	448 ± 6	[71]		cast, hom. at 500 ${}^{\circ }$C for 1 h, extr. to round bars (indirect w/o lubricant, 1:20) at 275 ${}^{\circ }$C with a die-exit speed of 24 m min${}^{-1}$, s.h.t. (500 ${}^{\circ }$C for 10 min) and w.q.
		T5	287 ± 2	306 ± 1	20 ± 2	194 ± 1	472 ± 3	[71]		as above, aged at 200 ${}^{\circ }$C for 0.5 h
	Mg-2.7Al-0.3Ca-0.4Mn	T4/s.h.t.	188 ± 1	267 ± 2	18 ± 1	121 ± 5	398 ± 4	[71]		cast, hom. at 500 ${}^{\circ }$C for 1 h, extr. to round bars (indirect w/o lubricant, 1:20) at 275 ${}^{\circ }$C with a die-exit speed of 24 m min${}^{-1}$, s.h.t. (500 ${}^{\circ }$C for 10 min) and w.q.
		T5	240 ± 2	283 ± 1	18 ± 1	149 ± 1	427 ± 3	[71]		as above, aged at 200 ${}^{\circ }$C for 0.5 h
**Mg-Zn-Ca**	Mg-0.2Zn-0.3Ca-0.1Mn	hom.	36	134	7.3			[85]		cast, cast, hom. at 400 ${}^{\circ }$C for 12 h and 450 ${}^{\circ }$C for 12 h, w.q.
		as-extr.	307		20.6			[85]		as above, extr. to round bars (indirect, 1:20) at 300 ${}^{\circ }$C with a die-exit speed of 1.2 m min${}^{-1}$
		as-extr.	164		30			[85]		as above, extr. to round bars (indirect, 1:20) at 350 ${}^{\circ }$C with a die-exit speed of 1.2 m min${}^{-1}$
	Mg-0.8Ca-0.37Zr	rolled, O, RD	133	196	14			[50]		cast, hom. at 300 ${}^{\circ }$C for 12 h w.q., rolled at 450 ${}^{\circ }$C from range 5 to 1 mm thickness in 4 passes using cold rollers, reheating for 6 min between passes, annealed at 400 ${}^{\circ }$C for 0.5 h
		rolled, O, TD	126	191	9			[50]		
		rolled, O, 45${}^{\circ }$	130	189	10			[50]		
	Mg-0.8Ca-0.37Zr-0.3Zn	rolled, O, RD	134	205	29			[50]		cast, hom. at 300 ${}^{\circ }$C for 12 h w.q., rolled at 450 ${}^{\circ }$C from range 5 to 1 mm thickness in 4 passes using cold rollers, reheating for 6 min between passes, annealed at 400 ${}^{\circ }$C for 0.5 h
		rolled, O, TD	104	198	34			[50]		
		rolled, O, 45${}^{\circ }$	110	200	32			[50]		
		rolled, T6	166	220	26			[50]		as above, aged in oil at 200 ${}^{\circ }$C for 1 h
		rolled, T8	201	226	20			[50]		as above, deformed to 0.015 plastic strain, aged in oil at 200 ${}^{\circ }$C for 1 h
	Mg-0.6Zn-0.6Ca-0.1Zr	as-rolled, RD	255 ± 2	276 ± 2	8			[31,32]		Twin-rolled cast (5.3 mm), rolled at range 370 to 400 ${}^{\circ }$C in 7 passes, with intermediate heating for 0.25 h to a thickness of 1.8 mm, cooled at air
		as-rolled, TD	200 ± 2	265 ± 2	23			[31,32]		
		as-rolled, 45${}^{\circ }$	217 ± 3	255 ± 2	19			[31,32]		
		rolled, O, RD	174 ± 3	239 ± 3	25			[31,32]		as above, annealed at 370 ${}^{\circ }$C for 0.5 h
		rolled, O, TD	135 ± 2	226 ± 2	31			[31,32]		
		rolled, O, 45${}^{\circ }$	152 ± 3	232 ± 2	27			[31,32]		
	Mg-0.7Zn-0.4Ca-0.1Mn	as-extr.	108	220	37.0			[84]		cast, hom. at 400 ${}^{\circ }$C for 12 h and 450 ${}^{\circ }$C for 12 h, w.q., extr. to round bars (indirect, 1:20) at 300 ${}^{\circ }$C with a die-exit speed of 6 m min${}^{-1}$
	Mg-0.8Zn-0.2Ca	rolled, O, RD	92.5 ± 2.5	182.5 ± 0.5	24 ± 1			[80]		cast, hom. at 400 ${}^{\circ }$C for 24 h, rolled (450 ${}^{\circ }$C) from range 5 to 1 mm in 8 passes, annealed at 400 ${}^{\circ }$C for 0.5 h
		rolled, O, TD	61 ± 1	186.5 ± 2	30.5 ± 0.5			[80]		
		rolled, O, 45${}^{\circ }$	68.5 ± 0.5	185 ± 2	32 ± 1			[80]		
	Mg-1.0Zn-0.2Ca	as-cast		125	8			[41]		cast, hom. at 430 ${}^{\circ }$C for 22 h and w.q.
		SPD		225	16			[41]		as above, preheated to pressing temp. for 0.3 h, ECAP (120${}^{\circ }$, 6mm min${}^{-1}$) for 2 passes at each temp. 400, 350 and 300 ${}^{\circ }$C
		SPD		263.7				[41]		ECAP as above, subseq. HPT (6GPa) for 0.5 revolutions at RT
		SPD		283.3				[41]		ECAP as above, subseq. HPT (6GPa) for 1 revolution at RT
	Mg-1.0Zn-0.3Ca	as-extr.	238	265	31	205		[28]		cast, hom. at 350 ${}^{\circ }$C for 12 h and 450 ${}^{\circ }$C for 8 h, cooled with pressurized air, aged at 250 ${}^{\circ }$C for 0.5 h, heated to extr. temp. (300 ${}^{\circ }$C) and held for 0.5 h, indirectly extr. to round bars (1:25) using a RAM speed of 0.5 mm s${}^{-1}$, cooled with pressurized air
		as-extr.	184	240	32	162		[28]		as above, heated to extr. temp. (325 ${}^{\circ }$C) and held for 0.5 h, indirectly extr. to round bars (1:25) using a RAM speed of 0.5 mm s${}^{-1}$, cooled with pressurized air
		as-extr.	247	268	20	184		[28]		as above, heated to extr. temp. (325 ${}^{\circ }$C) and held for 0.5 h, directly extr. to round bars (1:25) using a RAM speed of 0.5 mm s${}^{-1}$, cooled with pressurized air
		as-extr.	140	226	25	119		[28]		as above, heated to extr. temp. (400 ${}^{\circ }$C) and held for 0.5 h, directly extr. to round bars (1:25) using a RAM speed of 0.5 mm s${}^{-1}$, cooled with pressurized air
		as-extr.	240	255	27	205	245	[19]		as above, indirectly extr. to round bars (1:25) at 300 ${}^{\circ }$C using a RAM speed of 0.15 mm s${}^{-1}$
	Mg-1.0Zn-0.5Ca	as-cast	55 ± 2	120 ± 5	5 ± 0.5			[42]		cast in steel mold
		as-extr.	297 ± 2	300 ± 5	8 ± 1			[42]		cast, hom. at 400 ${}^{\circ }$C for 10 h, extr. (1:16) at 310 ${}^{\circ }$C, using an extr. speed of 4 mm s${}^{-1}$
		as-extr.	197 ± 3	256 ± 5	17 ± 1.5			[42]		as above, extr. (1:16) at 330 ${}^{\circ }$C, using an extr. speed of 4 mm s${}^{-1}$
		as-extr.	120 ± 3	200 ± 5	40 ± 3			[42]		as above, extr. (1:16) at 350 ${}^{\circ }$C, using an extr. speed of 4 mm s${}^{-1}$
		as-extr.	105 ± 3	205 ± 5	44 ± 3			[42]		as above, extr. (1:16) at 370 ${}^{\circ }$C, using an extr. speed of 4 mm s${}^{-1}$
		as-extr.	99 ± 3	201 ± 4	36 ± 2			[42]		as above, extr. (1:16) at 400 ${}^{\circ }$C, using an extr. speed of 4 mm s${}^{-1}$
		as-extr.	105	210	44			[60]		cast, extr. (1:16) at 350 ${}^{\circ }$C, using an extr. speed of 2 mm s${}^{-1}$
	Mg-1.0Zn-0.9Ca	rolled, T4	154.9	234.3	12.3			[81]		cast, hom. at 440 ${}^{\circ }$C for 1 h and w.q., hot rolled with 5 passes at 300 ${}^{\circ }$C to a overall reduction of 50%, intermediate reheating between the passes at 300 ${}^{\circ }$C for 0.3 h, annealing at 440 ${}^{\circ }$C for 0.5 h and w.q.
	Mg-1.2Zn-0.4Ca-0.2Zr	rolled, O, RD	229.6 ± 2.8	264.0 ± 2.2	24.7 ± 2.4			[82]		cast, hom. at 400 ${}^{\circ }$C for 4 h w.q., machined, rolled at 300 ${}^{\circ }$C (5 passes, with intermediate annealing, total reduction 72%), subseq. annealed at 300 ${}^{\circ }$C for 0.5 h
		rolled, O, 45${}^{\circ }$	290.8 ± 2.7	247.3 ± 0.2	32.3 ± 1.5			[82]		
		rolled, O, TD	171.3 ± 2.2	248.9 ± 2.5	27.5 ± 1.8			[82]		
	Mg-1.4Zn-0.1MM-0.1Zr	extr., O	200 ± 7	250 ± 5	15.3 ± 0.3	150 ± 6	441 ± 3	[83]		cast, pre-heated at 340 ${}^{\circ }$C, extr. (direct) to round bars at 300 ${}^{\circ }$C, air cooled, annealed at 300 ${}^{\circ }$C for 0.5 h
	Mg-1.4Zn-0.1MM-0.1Zr-0.4Ca	extr., O	171 ± 2	243 ± 1	14.6 ± 0.0	148 ± 1	432 ± 6	[83]		
	Mg-1.4Zn-0.1MM-0.1Zr-0.8Ca	extr., O	174 ± 1	243 ± 1	15 ± 1.1	149 ± 2	410 ± 4	[83]		
**Various**	Mg-1.8Mn-0.1Er	extr., O	173	255	7			[97]		cast, hom. at 450 ${}^{\circ }$C for 4 h air-cooled, machined, pre-heated to 450 ${}^{\circ }$C for 1 h, extr. to round bars (1:25) air-cooled, annealed at 390 ${}^{\circ }$C for 1 h
	Mg-1.8Mn-0.4Er	extr., O	224	276				[97]		
	Mg-1.8Mn-0.7Er	extr., O	228	275	12.5			[97]		
	Mg-1.8Mn-0.7Er-0.3Al	extr., O			19			[97]		
	Mg-0.3Sn-0.7Y	as-extr., ED	99.1 ± 1.0	264.2 ± 2.0	32.7 ± 0.2			[101]		cast, hom. at 400 ${}^{\circ }$C for 12 h, extr. (1:51) at 400 ${}^{\circ }$C, using a RAM speed of 3 mm s${}^{-1}$ to sheets
		as-extr., 45${}^{\circ }$	115.3 ± 0.5	226.8 ± 1.0	25.8 ± 0.3			[101]		
		as-extr., TD	124.9 ± 1.0	236.7 ± 1.0	24.7 ± 0.2			[101]		
		as-extr., ED	90.9 ± 0.8	191.9 ± 1.3	39.7 ± 0.3			[103,104]		
		as-extr., 45${}^{\circ }$	115.4 ± 1.1	182.5 ± 1.2	28.3 ± 0.3			[103,104]		
		as-extr., TD	119.6 ± 1.2	182.5 ± 1.2	28.3 ± 0.3			[103,104]		
		rolled, T4, RD	110.5 ± 1.3	199.5 ± 0.8	29.7 ± 0.3			[103]		cast, hom. at 400 ${}^{\circ }$C for 12 h, hot rolled with a mat. temp. of 400 ${}^{\circ }$C and roll temp. of 160 ${}^{\circ }$C, mat. reheated for 0.3 h after each pass, rolled to a sheet thickness of 1 mm with reductions of 20%, subseq. annealed at 400 ${}^{\circ }$C for 1 h and w.q.
		rolled, T4, 45${}^{\circ }$	109.9 ± 1.4	187.6 ± 0.7	34.7 ± 0.5			[103]		
		rolled, T4, TD	133.1 ± 0.9	184.5 ± 1.3	31.2 ± 0.1			[103]		

	Mg-0.4Sn-0.7Y-0.6Zn	as-extr., ED	188.4 ± 2.5	251.1 ± 1.4	33.1 ± 0.4			[104]		cast, hom. at 400 ${}^{\circ }$C for 12 h, extr. (1:51) at 400 ${}^{\circ }$C, using a RAM speed of 3 mm s${}^{-1}$ to sheets
		as-extr., 45${}^{\circ }$	130.3 ± 3.8	221.8 ± 1.9	47.2 ± 0.1			[104]		
		as-extr., TD	124.3 ± 2.0	231.4 ± 1.3	39.1 ± 0.2			[104]		
	Mg-0.5Sn-0.3Mn	as-extr., ED	130	239	10.4			[102]		cast, hom. at 400 ${}^{\circ }$C for 10 h air-cooled, machined, extr. to sheets at 400 ${}^{\circ }$C with an extr. speed of 1.2 m min${}^{-1}$
		as-extr., 45${}^{\circ }$	137	251	10			[102]		
		as-extr., TD	157	266	8.5			[102]		
	Mg-0.5Sn-0.3Mn-0.3Y	as-extr., ED	141	288	30.3			[102]		cast, hom. at 400 ${}^{\circ }$C for 10 h air-cooled, machined, extr. to sheets at 400 ${}^{\circ }$C with an extr. speed of 1.2 m min${}^{-1}$
		as-extr., 45${}^{\circ }$	170	286	28.1			[102]		
		as-extr., TD	188	302	28			[102]		
	Mg-1.1Sn-0.6Zn-0.5Ca	as-extr., ED	104.2 ± 1.8	311.9 ± 2.4	30.5 ± 1.4			[100]		cast, hom. at 400 ${}^{\circ }$C for 12 h, extr. (1:32) at 400 ${}^{\circ }$C, using a RAM speed of 1 mm s${}^{-1}$ to sheets with a thickness of 3 mm
		as-extr., TD	188.9 ± 2.5	295.6 ± 3.4	12.9 ± 0.4			[100]		
	Mg-1.2Sn-0.5Zn	as-extr., ED	129.4 ± 2.4	260.9 ± 2.7	17.6 ± 0.9			[100]		cast, hom. at 400 ${}^{\circ }$C for 12 h, extr. (1:32) at 400 ${}^{\circ }$C, using a RAM speed of 1 mm s${}^{-1}$ to sheets with a thickness of 3 mm
		as-extr., TD	153.1 ± 1.2	262.1 ± 1.9	14.3 ± 0.6			[100]		
	Mg-1.3Sn-0.7Ca	as-extr., ED	137.8 ± 3.2	264.8 ± 3.2	17.3 ± 1.1			[100]		cast, hom. at 400 ${}^{\circ }$C for 12 h, extr. (1:32) at 400 ${}^{\circ }$C, using a RAM speed of 1 mm s${}^{-1}$ to sheets with a thickness of 3 mm
		as-extr., TD	209.3 ± 3.5	293.7 ± 3.2	10.3 ± 0.5			[100]		
	Mg-0.5Zn-0.2Ca	as-extr.	119 ± 2	224 ± 1	25 ± 2	93 ± 1	331 ± 3	[88]		cast, hom. at 370 ${}^{\circ }$C for 24 h and quenched in warm water, preheated to 375 ${}^{\circ }$C for 1 h, indirectly extr. (1:25) at 375 ${}^{\circ }$C, using an extr. speed of 2.2 mm s${}^{-1}$ to round bars
	Mg-0.5Zn-0.2Ge	as-extr.	171 ± 2	249 ± 2	10 ± 1	75 ± 1	367 ± 6	[88]		cast, hom. at 320 ${}^{\circ }$C for 24 h and quenched in warm water, preheated to 375 ${}^{\circ }$C for 1 h, indirectly extr. (1:25) at 375 ${}^{\circ }$C, using an extr. speed of 2.2 mm s${}^{-1}$ to round bars
	Mg-0.6Zn-0.3Zr-0.6Nd	rolled, O, RD	111	243	27.58			[89]		cast, hom. at 450 ${}^{\circ }$C for 12 h and w.q., hot rolled at 400 ${}^{\circ }$C from range 8 to 2 mm at a reduction of range 15 to 20% per pass, intermediate reheating between the passes at 400 ${}^{\circ }$C for 10 min, subseq. w.q., annealing at 440 ${}^{\circ }$C for 1 h
		rolled, O, 45${}^{\circ }$	101	234	27.95			[89]		
		rolled, O, TD	84	238	28.61			[89]		
	Mg-0.7Zn-0.2Zr-0.8Ce	rolled, O, RD	131	234	16.86			[89]		
		rolled, O, 45${}^{\circ }$	110	237	22.96			[89]		
		rolled, O, TD	115	222	12.61			[89]		
	Mg-0.7Zn-0.2Zr-0.7Gd	rolled, O, RD	88	229	29.33			[89]		
		rolled, O, 45${}^{\circ }$	78	227	29.93			[89]		
		rolled, O, TD	66	233	32.19			[89]		
	Mg-0.8Zn-0.3Zr-0.9MM	rolled, O, RD	131	233	18.95			[89]		
		rolled, O, 45${}^{\circ }$	108	243	25.27			[89]		
		rolled, O, TD	99	247	24.86			[89]		
	Mg-0.9Zn-0.2Zr-0.7La	rolled, O, RD	123	241	22.75			[89]		
		rolled, O, 45${}^{\circ }$	108	240	24.78			[89]		
		rolled, O, TD	96	243	25.34			[89]		
	Mg-1.0Zn-0.4Zr	as-extr.	237	271	24	165	436	[93]		cast, hom. at 350 ${}^{\circ }$C for 15 h, extr. to round bars (indir., 1:30) at 300 ${}^{\circ }$C, using a speed of 1 m min${}^{-1}$
		as-extr.	196	248	19	96	370	[93]		as above, using a speed of 5 m min${}^{-1}$
		as-extr.	188	245	21	101	366	[93]		as above, using a speed of 10 m min${}^{-1}$
	Mg-1.0Zn-0.4Zr-0.8MM	as-extr.	296	299	18	184	434	[93]		cast, hom. at 350 ${}^{\circ }$C for 15 h, extr. to round bars (indir., 1:30) at 300 ${}^{\circ }$C, using a speed of 1 m min${}^{-1}$
		as-extr.	221	260	21	156	381	[93]		as above, using a speed of 5 m min${}^{-1}$
		as-extr.	201	251	19	142	369	[93]		as above, using a speed of 10 m min${}^{-1}$
	Mg-1.2Zn-0.2Zr	rolled, O, RD	211.6 ± 2.3	247.4 ± 2.7	19.8 ± 1.6			[82]		cast, hom. at 400 ${}^{\circ }$C for 4 h w.q., machined, rolled at 300 ${}^{\circ }$C (5 passes, with intermediate annealing, total reduction 72%), subseq. annealed at 300 ${}^{\circ }$C for 0.5 h
		rolled, O, 45${}^{\circ }$	204.3 ± 0.5	242.6 ± 1.6	25.9 ± 1.3			[82]		
		rolled, O, TD	203.1 ± 2.5	243.5 ± 3.4	21.2 ± 0.8			[82]		
	Mg-1.3Zn-0.1Ce	as-extr.	206 ± 1	261 ± 3	20.1 ± 0.4	124 ± 2	410 ± 7	[92]		cast, extr. to round bars (indir., 1:30) at 300 ${}^{\circ }$C, using a speed of 1 m min${}^{-1}$
		as-extr.	176 ± 2	245 ± 2	11.7 ± 2.5	81 ± 1	361 ± 4	[92]		as above, using a speed of 10 m min${}^{-1}$
		as-extr.	156 ± 2	226 ± 2	18.1 ± 1.3	70 ± 1	347 ± 1	[92]		as above, using a speed of 20 m min${}^{-1}$
	Mg-1.3Zn-0.2Ce-0.5Zr	as-extr.	305 ± 3	313 ± 3	21.0 ± 4.0	173 ± 1	436 ± 15	[92]		cast, extr. to round bars (indir., 1:30) at 300 ${}^{\circ }$C, using a speed of 1 m min${}^{-1}$
		as-extr.	204 ± 1	257 ± 1	20.3 ± 0.9	126 ± 4	384 ± 13	[92]		as above, using a speed of 10 m min${}^{-1}$
		as-extr.	209 ± 2	259 ± 2	22.6 ± 0.5	124 ± 1	375 ± 3	[92]		as above, using a speed of 20 m min${}^{-1}$
	Mg-1.6Zn-0.5Gd	as-extr.	117	213	30.1			[90]		cast, hom. at 480 ${}^{\circ }$C for 12 h, indirectly extr. (1:20) at 300 ${}^{\circ }$C with a die-exit speed of 6 m min${}^{-1}$ to round bars
		as-extr.	283	295	10	154		[43]		cast, hom. at 480 ${}^{\circ }$C for 12 h, indirectly extr. (1:20) at 400 ${}^{\circ }$C with a die-exit speed of 1.2 m min${}^{-1}$ to round bars
		as-extr.	161	233	24.7	111		[43]		cast, hom. at 480 ${}^{\circ }$C for 12 h, indirectly extr. (1:20) at 350 ${}^{\circ }$C with a die-exit speed of 1.2 m min${}^{-1}$ to round bars
		as-extr.	254	284	27.9	190		[43]		cast, hom. at 480 ${}^{\circ }$C for 12 h, MDIF at 300 ${}^{\circ }$C, w.q., indir. extr. (1:20, 350 ${}^{\circ }$C) at a die-exit speed of 1.2 m min${}^{-1}$ to round bars
		as-extr.	181	248	34.8	134		[43]		MDIF as above, w.q., indirectly extr. (1:20) at 400 ${}^{\circ }$C with a die-exit speed of 1.2 m min${}^{-1}$ to round bars
	Mg-1.8Zn-0.1Nd-0.1Ce	as-rolled, RD	270 ± 2	288 ± 2	19			[32]		Twin-rolled cast (5.3 mm), rolled at range 370 to 400 ${}^{\circ }$C in 7 passes, with intermediate heating for 0.25 h to a thickness of 1.8 mm, cooled at air
	-0.05La-0.2Y	as-rolled, TD	190 ± 5	266 ± 2	20			[32]		
		as-rolled, 45${}^{\circ }$	197 ± 3	250 ± 2	26			[32]		
		rolled, O, RD	181 ± 4	244 ± 4	33			[32]		as above, annealed at 350 ${}^{\circ }$C for 0.5 h
		rolled, O, TD	106 ± 3	221 ± 4	35			[32]		
		rolled, O, 45${}^{\circ }$	118 ± 4	215 ± 3	40			[32]		
		as-rolled, RD	298 ± 2	320 ± 4	16			[32]		Twin-rolled cast (5.3 mm), rolled at range 370 to 400 ${}^{\circ }$C in 7 passes, with intermediate heating for 0.25 h to a thickness of 1.8 mm, cooled at air subseq. cold rolled (2 passes, 0.1 true strain)
		as-rolled, TD	205 ± 3	286 ± 2	27			[32]		
		as-rolled, 45${}^{\circ }$	220 ± 3	282 ± 2	33			[32]		
		rolled, O, RD	197 ± 3	257 ± 2	33			[32]		as above, annealed at 350 ${}^{\circ }$C for 0.5 h
		rolled, O, TD	121 ± 2	232 ± 3	42			[32]		
		rolled, O, 45${}^{\circ }$	134 ± 3	231 ± 2	43			[32]		
